# Real-time prediction of HFNC treatment failure in acute hypoxemic respiratory failure using machine learning

**DOI:** 10.1038/s41598-025-16061-x

**Published:** 2025-08-18

**Authors:** Xiaojie Li, Chunliang Jiang, Qingyan Xie, Huiquan Wang, Jiameng Xu, Guanjun Liu, Panpan Chang, Guang Zhang

**Affiliations:** 1 School of Life Sciences, Tiangong University, Tianjin, 300387 China; 2 School of Control Science and Engineering, Tiangong University, Tianjin, 300387, China; 3 Systems Engineering Institute, Academy of Military Sciences, People’s Liberation Army, Tianjin, 300161 China; 4 Trauma Medicine Center of Peking University People’s Hospital, Key Laboratory of Trauma and Neural Regeneration (Peking University) Ministry of Education, National Center for Trauma Medicine of China, Beijing, 100044, China

**Keywords:** High-flow nasal cannula, Acute hypoxemic respiratory failure, Machine learning methods, Real-time dynamic alert, ROX index, mROX index, Bioinformatics, Data mining, Data processing, Machine learning

## Abstract

Accurate and timely prediction of high-flow nasal cannula (HFNC) treatment failure in patients with acute hypoxemic respiratory failure (AHRF) can lower patient mortality. Previous studies have highlighted inconsistencies in the predictive performance of existing indices, such as ROX and mROX, which are limited by their reliance on oxygenation parameters alone. To address this, we developed a machine learning-based predictive model using temporal data from AHRF patients, aimed at facilitating quicker development of individualized treatment plans and intervention strategies for healthcare professionals. We extracted 15 non-invasive and 15 laboratory features, including patient demographic characteristics, Glasgow Coma Scale, blood gas analysis, chemical assay, and complete blood cell count features. In addition to five machine learning models and an ensemble classifier, an long short-term memory (LSTM) network was included to assess deep learning performance on time-series data. Our study enrolled 427 patients with 498 treatment records. The soft-voting ensemble algorithm achieved an optimal predictive performance with an AUC of 0.839 (95% CI 0.786–0.889) for the all-features model, while logistic regression using common features achieved an AUC of 0.767 (95% CI 0.704–0.825), outperforming ROX and mROX indices. Incorporating blood gas analysis features improved the non-invasive model’s performance by 0.104. This study introduces a machine learning model integrated with a dynamic real-time alert system for predicting HFNC treatment failure in AHRF patients, demonstrating improved performance over traditional indices in internal validation and showing potential for decision support in select healthcare settings.

## Introduction

Acute hypoxemic respiratory failure (AHRF), or type 1 respiratory failure, is defined by hypoxemia without hypercapnia, primarily caused by impaired pulmonary oxygenation^1^. Common etiologies include pneumonia, non-cardiogenic pulmonary edema, ARDS, and ventilation-perfusion mismatch. If untreated, AHRF can progress to tissue hypoxia, multi-organ failure, and death.

High-flow nasal cannula (HFNC) is increasingly used to manage AHRF, offering high flow rates and humidified oxygen that reduce airway dryness and enhance patient comfort^2–5^. Compared to conventional oxygen therapy (COT), HFNC improves oxygenation and lung compliance, and has been associated with lower reintubation rates^6^.

Invasive mechanical ventilation (IMV) is a therapeutic method that delivers air or oxygen to a patient’s lungs through intubation, either assisting or replacing their spontaneous breathing. This technique is widely used in critical care settings to support respiratory function, ensuring sufficient oxygenation and elimination of carbon dioxide. As such, IMV is pivotal in the management of patients with respiratory dysfunction^7–9^.

HFNC treatment failure often leads to delayed intubation, resulting in various adverse clinical outcomes^10^. Kirsten et al. demonstrated^11,12^ that the mortality rate of late-stage IMV patients is significantly higher than that of early-stage IMV patients and patients who do not require IMV. Kang et al.^10^ further explored this risk and reported that the mortality rate of intubation after 48 h of HFNC treatment was 27.5% higher than that within 48 h of intubation. This pivotal study highlights the risk of delayed intubation. Ricard et al.^13^, identified several reasons that may explain this risk: Prolonged periods of patient-initiated breathing can exacerbate the initial injury, a phenomenon referred to as patient-induced lung injury. In addition, HFNC may mask clinical deterioration by normalizing oxygen saturation, even when underlying problems such as ventilation-perfusion mismatch or alveolar hypoventilation requiring pressure support remain uncorrected. This can potentially delay necessary interventions^14^. As a result, the patient’s condition may be silently deteriorating, leading to respiratory muscle fatigue, and even triggering cardiac dysfunction and organ failure, thereby impacting prognosis. Therefore, close monitoring is essential for patients receiving HFNC treatment to promptly detect clinical deterioration and avoid delayed intubation.

Junhai et al.^15^ conducted a systematic review and meta-analysis on the use of the ROX index for failure of HFNC treatment prediction. The study revealed a lack of universal consensus on the critical value of ROX. Within the first 12 h of HFNC treatment, no significant change was detected in the trend of the ROX index. Li et al.^16^ utilized the arterial partial pressure of oxygen (PO2)-corrected mROX index, as well as the mROX-HR index, which incorporates heart rate, to predict the failure of HFNC treatment. Despite more accurate predictive performance of the mROX and mROX-HR indices, PO2, which is used as a laboratory feature, requires arterial blood gas analysis, limiting its applicability in pre-hospital scenarios. Goh et al.^17^ evaluated a modified ROX index that included heart rate (ROX-HR) and reported that the optimal threshold of ROX-HR at different time points could not be determined. On the other hand, Gallardo et al.^18^ proposed that the ROX index can be considered a static indicator that may not effectively reflect changes in a patient’s condition over time.

In recent years, the application of machine learning and deep learning in the field of medicine and healthcare has rapidly expanded^19–21^. Therefore, this study proposes a real-time early warning key technology for HFNC treatment failure in AHRF patients. The aims of this study are as follows: First, to develop a dynamic real-time warning model that enables timely professional intervention after HFNC treatment failure; second, to investigate the potential of using only common features for early warning, aligning with clinical practice requirements for user-friendliness and timeliness in various scenarios; finally, to conduct interpretability analysis on relevant input features, optimizing the feature set for efficient and wider application.

## Methods

**Fig. 1:**
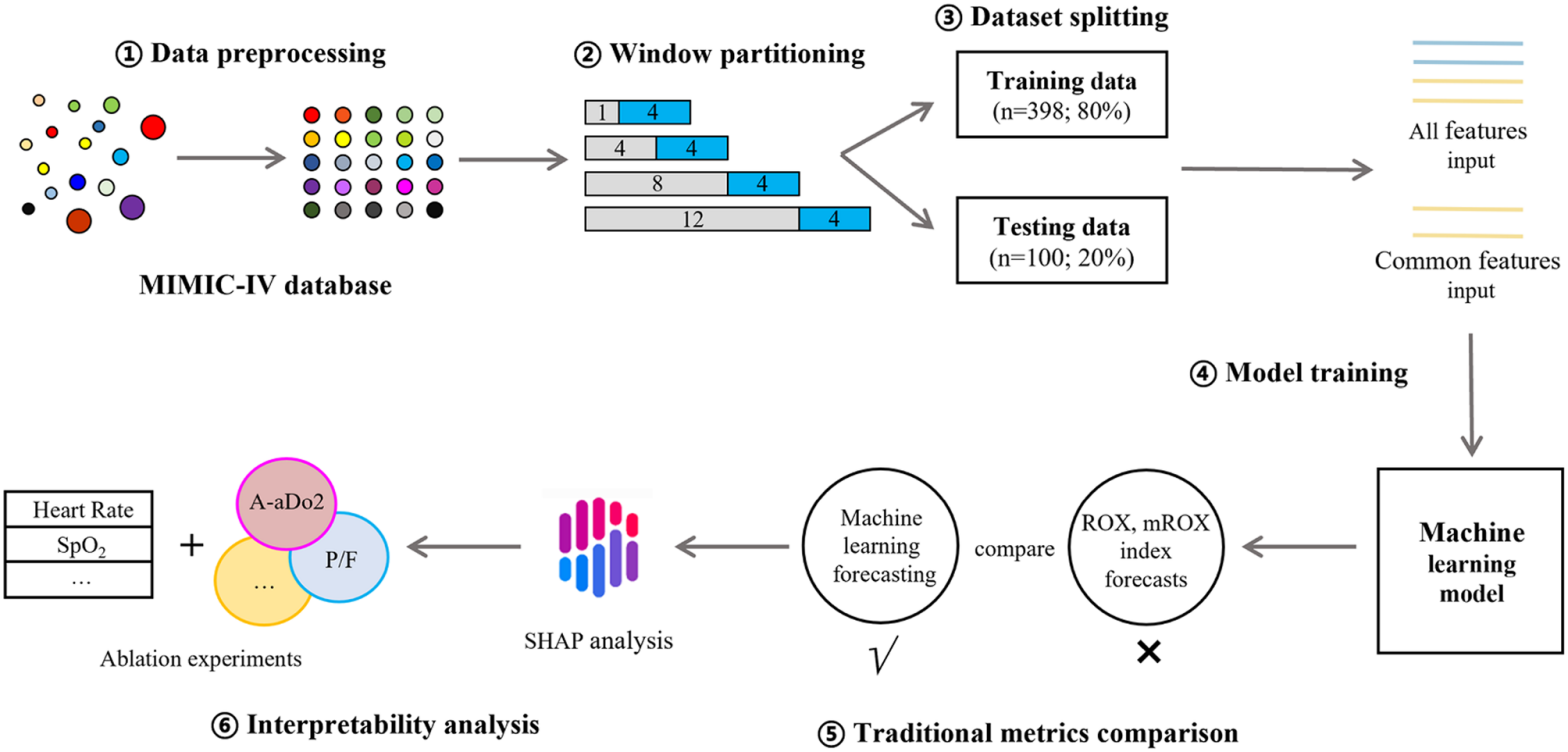
The study flowchart. Data from the MIMIC-IV database, with a total of 427 eligible patients was used. Among them, 393 instances of successful HFNC treatment and 105 instances of treatment failure were allocated into training and testing sets.

Figure 1 illustrates the design and flowchart employed in this study:

1. The physiological data obtained from MIMIC-IV was preprocessed.
2. Four combinations of dynamic observation-prediction windows were defined.
3. The dataset was divided into training and testing sets with an 8:2 ratio.
4. Machine learning models were trained using all features and common features as inputs.
5. The methods presented in this study were compared to two traditional metrics (ROX index, mROX index).
6. Interpretability analysis and ablation experiments were performed using SHAP algorithm.

### Source of data

The dataset utilized in this retrospective study was extracted by the MIMIC-IV database^22^. The MIMIC database was established in 2003 with funding from the National Institutes of Health (NIH) and is a collaborative effort involving the Laboratory for Computational Physiology at MIT, Beth Israel Deaconess Medical Center (BIDMC), and Philips Healthcare. Clinical data from over 190,000 patients and approximately 450,000 hospital admissions at BIDMC between 2008 and 2019 are included in the database. Demographic information, laboratory results, medication records, vital signs, surgical procedures, disease diagnoses, medication management, follow-up and survival status, and other detailed patient information is available.

### Participants and eligibility criteria

AHRF is defined as the ratio of arterial partial pressure of oxygen (PaO_2_) to the fraction of inspired oxygen (FiO_2_) ≤ 300 mmHg^6,23,24^, and is not accompanied by hypercapnia^23^.

Although the 2023 ATS/ESICM criteria allow for the diagnosis of ARDS without the requirement for invasive mechanical ventilation, the absence of systematically recorded imaging findings (e.g., chest radiography or CT) and echocardiographic data to exclude cardiogenic pulmonary edema in the MIMIC-IV database precluded a definitive ARDS diagnosis. Therefore, we adopted the broader term acute hypoxemic respiratory failure (AHRF) defined as PaO_2_/FiO_2_ ≤ 300 mmHg without hypercapnia as a practical surrogate for cohort selection. This approach ensures consistency with the available data while still encompassing a large proportion of patients who likely fulfill the criteria for non-intubated ARDS^25^.

HFNC treatment success is defined as a downgrade of respiratory support to COT or spontaneous breathing after weaning from HFNC. HFNC treatment failure is defined as an upgrade of respiratory support to IMV or tracheostomy within one hour of weaning from HFNC.

In this study, various observation windows (1, 4, 8, and 12 h) were introduced to investigate the impact of observation duration on model performance in detail. Specifically, the 12-h window aligns with the recommended HFNC treatment duration^26^, where extending the observation period beyond 12 h may adversely affect early detection and timely intervention, increasing the risk of delayed intubation. Conversely, the 1-h window allows for more immediate prediction capability, while the 4-h and 8-h windows provide intermediate intervals between the shortest and longest durations, designed to explore how a gradual increase in window length affects model accuracy. These various observation windows not only balances the real-time responsiveness and accuracy of predictions but also aligns with clinical practices of evaluating HFNC efficacy, offering healthcare providers a range of decision-support options.

Observation window: The observation window was set to 1, 4, 8, and 12 h, respectively. The failure of HFNC treatment in patients with AHRF was predicted using the data within this window.

Prediction window: The prediction window was used to determine whether HFNC treatment failure occurred within this window period. Considering the demand for real-time prediction and clinical practice experience, a 4-h prediction window was used^27,28^.

Rationale for the window design: Clinically, the observation window reflects the period during which a physician would monitor patient vital signs and trends to form a clinical judgment, whereas the prediction window represents a realistic timeframe in which proactive interventions could be initiated if deterioration is anticipated.

Common features: physiological and clinical parameters obtained without requiring arterial catheterization, blood sampling, or other invasive procedures.

**Fig. 2:**
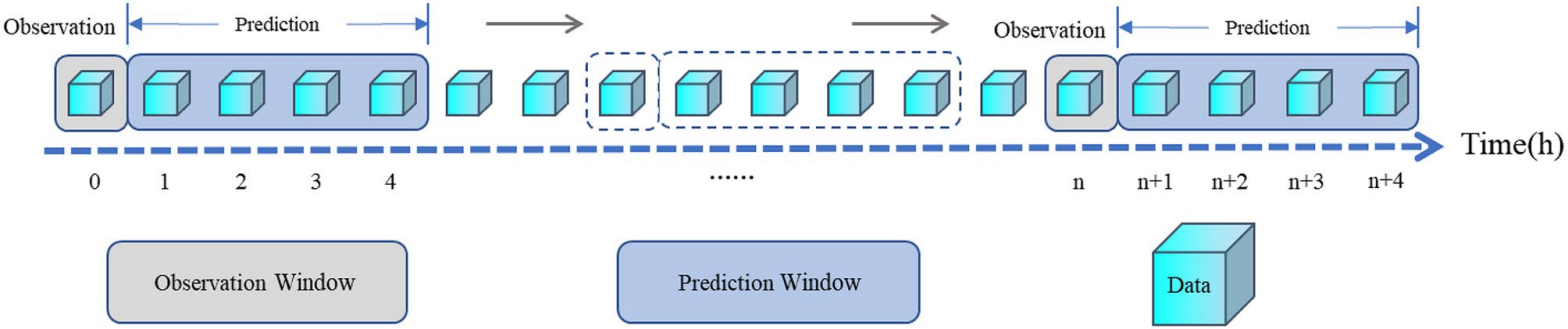
Implementation diagram of the dynamic window. The combination of set observation window and prediction window slides every hour along the timeline.

Figure 2 and (Appendix 1 (Supplementary Fig. 1)) shows the combination of a 1-h observation window and a 4-h prediction window as an example to illustrate the real-time dynamic warning window.

The following criteria were used to enroll patients into the study:

- Adult patients (age ≥ 18 years).
- Diagnosed with AHRF.
- Undergoing HFNC treatment.
- HFNC treatment duration between 12 and 48 h.

**Fig. 3:**
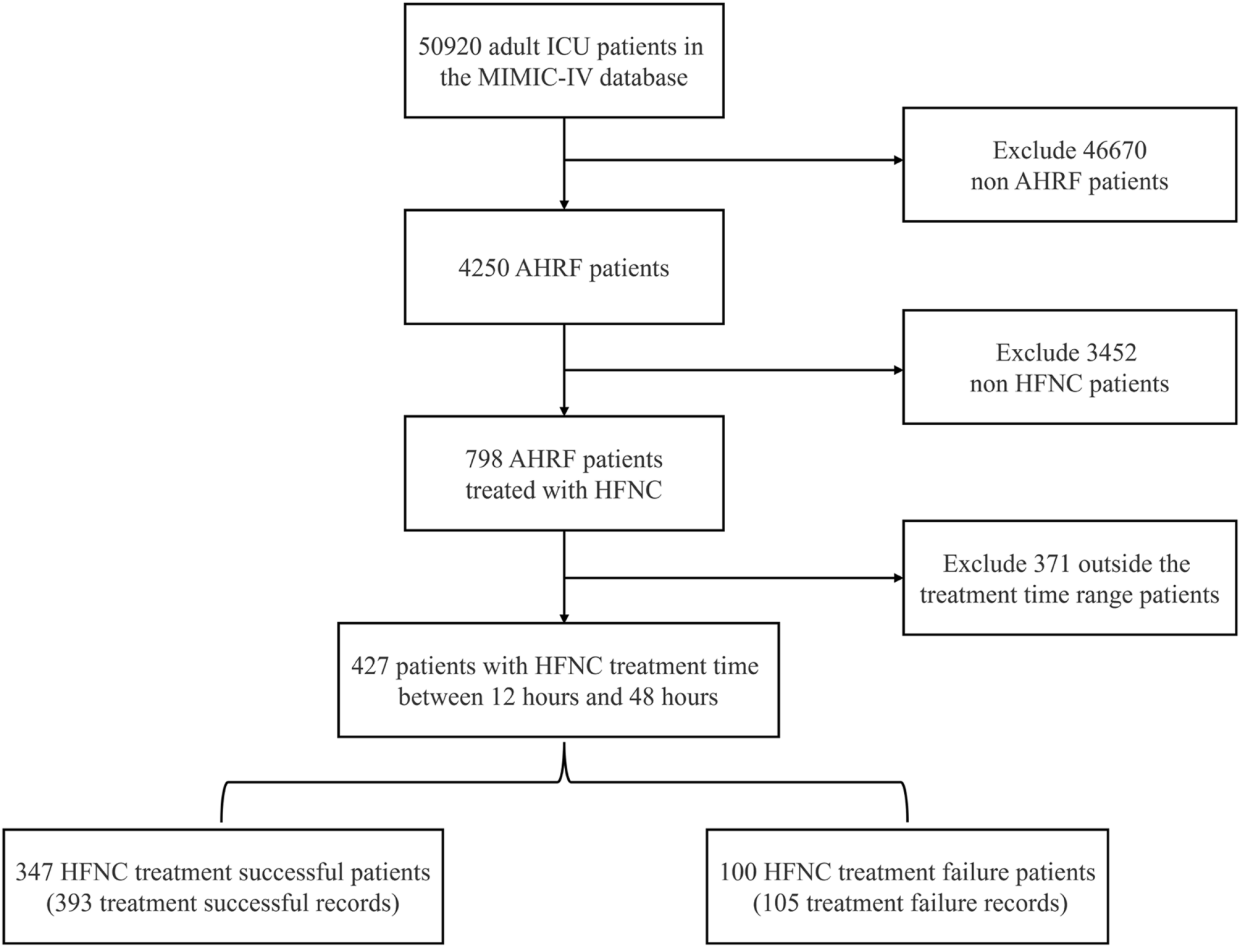
Process for selection of the cohort.

The detailed process of data selection is shown in Fig. 3.

Based on the aforementioned four criteria, a total of 427 patients were included. There were 498 instances of HFNC treatment recorded, including 393 instances of successful treatment and 105 instances of treatment failure. Each recorded HFNC treatment instance in this study was treated as an independent sample for analysis.

### Outcome definition and predictors

A total of 30 features were extracted from the MIMIC-IV database. The common features included demographic features, physiological features and Glasgow Coma Scale score. The laboratory features comprised blood gas analysis, chemistry assay, and complete blood cell count. The selection of these features was informed not only by our previous work and related studies on intubation prediction, but also by their clinical relevance in capturing the patient’s overall physiological status and oxygenation dynamics. Especially during HFNC treatment, these features dynamically capture changes in respiratory function and metabolic condition, providing critical inputs for the model to predict patients’ responses to treatment. The hourly median of numerical data was used, with a sampling frequency of 1 h. Table 1 presents the selected features and their types.

**Table 1:** The 30 features that were extracted from the MIMIC-IV database. The full names of all features can be found in Appendix 1 (Supplementary Table 6).

	Category	Features
Common features	Demographic features	Age, BMI, gender
	Physiological features	Urine output, heart rate, non-invasive systolic blood pressure, non-invasive diastolic blood pressure, non-invasive mean blood pressure, respiration rate, temperature, SpO_2_
	Glasgow scoring	Gcs, Gcs verbal, Gcs motor, Gcs eyes
Laboratory features	Blood gas analysis features	Oxygenation index, A-ado2, Base excess, TotalCO2, PCO2, PH, PO2
	Chemical assay features	Creatinine, Glucose, Bun
	Complete blood cell count features	Hematocrit, hemoglobin, platelet, Rbc, Wbc

### Data preprocessing and handling of missing data

#### Data standardization

Data standardization was performed using the Z-score method^29^. Z-score normalization was used to scale each feature to a standard normal distribution with a mean of zero and a standard deviation of one. This normalization method eliminates the scale differences between different features, making the feature weights more consistent and facilitating model convergence.

#### Missing value imputation

The following imputation methods were used for physiological features: For urine output, if the patient had a null value for a particular hour, it was assumed that there was no urine output during that hour, and thus it was imputed with zero. For other physiological features, the previous value was used for imputation. If there was no previous value, the global median was used instead.

#### One-hot encoding

One-hot encoding is a widely used technique often employed to convert categorical variables into a format suitable for machine learning methods. In one-hot encoding, each value of a categorical variable is transformed into a binary vector of length equal to the number of possible values of that variable. Only one element in the binary vector is set to 1, while the others are set to 0^30^. The position corresponding to the specific value of a variable is marked as 1. In this study, one-hot encoding was used to process the gender variable.

#### Data imbalance issue

To address the issue of data imbalance, this study employed a "pseudo-over-sampling" method for data balancing. Specifically, for each patient, the number of virtual data rows equal to the length of the prediction window was added—in this study, 4 rows of virtual data were added for each patient. These 4 rows of data were identical to the last row of the patient’s real data. When the dynamic window slides to the maximum extent in the time series, the prediction window exactly occupies the 4 rows of virtual data, while the observation window slides to the last hour of the real data. The advantages of this method are as follows:

(1) The amount of positive data was increased, expanding the number of positive data rows by 4 times; (2) During model training, only the binary classification labels of the virtual data were used, without incorporating the virtual data itself into model training; (3) The observation window only included all real data.

### Model development

In this study, five machine learning methods and a soft voting ensemble machine learning algorithm were employed, and the results were compared to the ROX and mROX indices. To further enhance the predictive performance of these models, a custom grid search method incorporating an optimal classification threshold algorithm was employed for hyperparameter optimization. Taking the LGBM algorithm as an example, the primary optimized hyperparameter settings are as follows: the number of trees (n_estimators), tree depth (max_depth), and number of leaves (num_leaves) are set to 100, 10, and 31, respectively.

Logistic regression (LR)^31^: A linear model used in binary classification problems by transforming the linear combination of input features into probability outputs using a logistic function.

Naive Bayes (NB)^32^: Based on Bayes’ theorem and the assumption of conditional independence between features, this model works well with high-dimensional data but performs poorly in the presence of feature dependencies.

Support vector machine (SVM)^33^: A supervised learning algorithm used for classification and regression tasks, which calculates an optimal hyperplane in a high-dimensional space to maximize the margins between categories, thus achieving data classification.

Random forest (RF)^34^: An ensemble learning model composed of multiple decision trees that improves the accuracy of classification or regression by using majority voting or the mean prediction of the individual decision trees for classification and regression, respectively.

LGBM (LightGBM)^35^: A decision tree algorithm based on the gradient boosting framework, which accelerates the training process using histogram-based algorithms, suitable for large-scale datasets.

Long Short-Term Memory (LSTM)^36^:A type of recurrent neural network (RNN) architecture designed to capture long-term dependencies in sequential data by using memory cells and gating mechanisms. It is particularly well-suited for modeling time-series data, such as physiological signals, where temporal dynamics play a crucial role in prediction tasks.

**Fig. 4:**
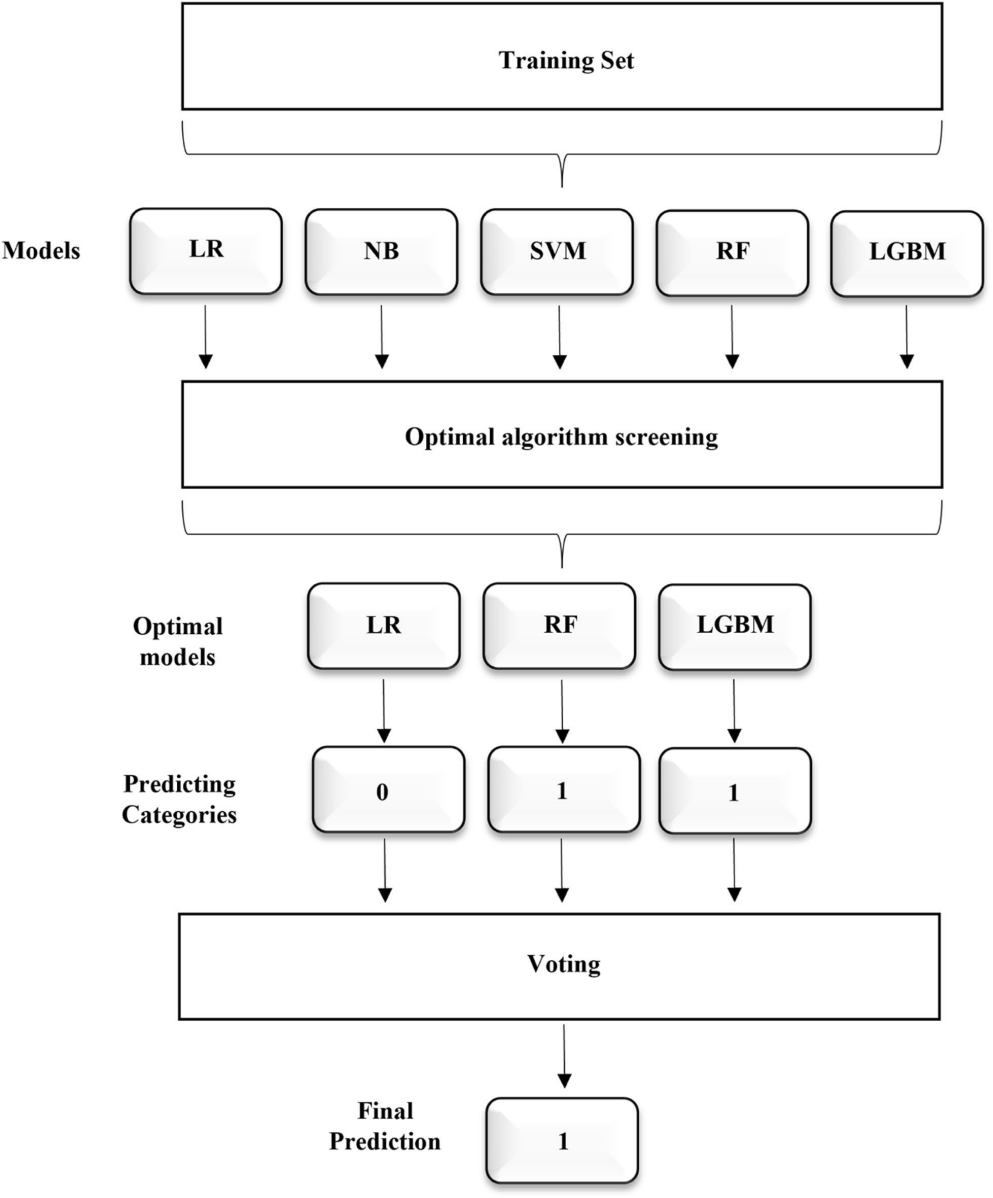
The principle of soft voting ensemble machine learning algorithms. *LR* Logistic regression, *NB* Naive Bayes, *SVM* Support vector machine, *RF* Random forest, *LGBM* LightGBM.

Soft voting ensemble machine learning algorithm^37^: The soft voting ensemble machine learning method is an ensemble learning technique that aggregates the probability predictions of multiple base models to make decisions, thereby improving classification or regression accuracy. Figure 4 illustrates the principle of the soft voting ensemble machine learning algorithm. In this study, three models (LR, RF, LGBM) with smaller differences in sensitivity and specificity and higher AUC values were used in the soft voting ensemble.

LR, NB, SVM, RF, LGBM, and LSTM represent classic algorithms in the fields of statistics, probability theory, geometry, decision tree modeling, gradient boosting, and deep learning, respectively. The soft voting ensemble machine learning algorithm enhances the performance and stability of the overall model by aggregating the prediction results of multiple base learners, so as to achieve more accurate and reliable predictions.

### Model performance and validation

#### Traditional prediction methods

ROX Index: The ROX index threshold of 4.88 was adopted to predict HFNC treatment failure, whereby values below 4.88 were interpreted as likely treatment failure and values equal to or above 4.88 as likely treatment success. This threshold is broader than the commonly used cutoff of 3.85 reported in previous studies, and was intended to allow for a more inclusive identification of patients at high risk of treatment failure^38,39^.

mROX Index^16^: The mROX index is an improvement of the ROX index, where SpO_2_ is replaced with PO_2_ to more accurately reflect oxygenation status. Li^16^ evaluated the predictive effect of the mROX index at the 2nd hour after initiating HFNC. Therefore, in this study, an mROX index less than 4.3 at the 2nd hour after initiating HFNC was used to predict treatment failure, based upon previous research.

#### Performance evaluation metrics

The following metrics were used to evaluate the performance of the machine learning models: Accuracy (ACC), area under the curve (AUC) of the receiver operating characteristic (ROC) curve, sensitivity (SEN), specificity (SPE), Bayesian error rate (BER), Matthews correlation coefficient (MCC), F1-score, and KAPPA^40^. Table 2 presents the calculation formulas and brief descriptions of these eight metrics.

**Table 2:** Description and formula of performance indicators.

Metric	Formula	Description
ACC	$\frac{TP}{TP+FN+FP+FN}$	The proportion of correctly classified samples to the total sample size
AUC	$\frac{{\sum }_{i=1}^{N}\left(TP{R}_{i}+TP{R}_{i-1}\right)\cdot \left(FP{R}_{i}-FP{R}_{i-1}\right)}{2}$	The area under the Receiver Operating Characteristic (ROC) curve
SEN	$\frac{TP}{TP+FN}$	The proportion of samples correctly classified as positives to all positive samples
SPE	$\frac{TN}{TN+FP}$	The proportion of samples correctly classified as negatives to all negative samples
BER	$\frac{1}{2}\cdot \left(\frac{FP}{FP+TN}+\frac{FN}{FN+TP}\right)$	The average misclassification rate of the classifier considering the weights of positive and negative samples
MCC	$\frac{TP\cdot TN-FP\cdot FN}{\sqrt{\left(TP+FP\right)\left(TP+FN\right)\left(TN+FP\right)\left(TN+FN\right)}}$	A metric considering true positives, true negatives, false positives, and false negatives to comprehensively evaluate classifier performance
F1-score	$2\cdot \frac{\left(TP/\left(TP+FP\right)\right)\cdot \left(TP/(TP+FN)\right)}{\left(TP/(TP+FP)\right)+\left(TP/(TP+FN)\right)}$	The harmonic mean of precision and recall
KAPPA	$\frac{{P}_{o}-{P}_{e}}{1-{P}_{e}}$	A statistical measure to assess the consistency between classifiers or evaluators

### Internal validation

Temporal internal validation was performed by training the model on data from 2008 to 2013 and testing on data from 2014 to 2019, using a 1-h observation window and a 4-h prediction window.

### Model explainability analysis

#### SHAP-based interpretation of feature contributions

To enhance interpretability, SHapley Additive exPlanations (SHAP) were applied to quantify the contribution of each predictor to the model output. SHAP values represent the marginal contribution of individual features to a given prediction, derived from cooperative game theory. This approach allows for both global interpretation by summarizing the average impact of features across the dataset and local interpretation, which reveals how specific features influence predictions for individual patients.

#### Feature selection using recursive elimination

Recursive feature elimination with cross-validation (RFECV) was performed to evaluate the trade-off between model complexity and predictive performance. This technique iteratively eliminated the least important features based on model performance metrics, resulting in a parsimonious subset of predictors. The selected features were subsequently used for final model development to optimize both performance and interpretability.

#### Calibration analysis for clinical reliability

Model calibration was assessed using the Brier score, which quantifies the mean squared difference between predicted probabilities and actual outcomes. Lower Brier scores indicate better calibration. Comparative calibration analysis across all candidate models demonstrated that the ensemble model consistently achieved the lowest Brier scores, suggesting a higher degree of reliability in predicted probabilities.

### Software and reproducibility

All data preprocessing, feature selection, model training, and interpretability analyses were conducted using Python 3.12 in a Jupyter Notebook environment. The computational workflow was based on open-source packages including scikit-learn, LightGBM, SHAP, XGBoost, pandas, and NumPy. Model development and visualization were performed interactively in Jupyter Notebook. The source data were stored and queried from a PostgreSQL 15.3 database, which was used for efficient data extraction and time window alignment from the MIMIC-IV relational database.

## Results

### Baseline characteristics of patient treatment records

According to the enrollment criteria listed in "Data preprocessing and handling of missing data", a total of 498 treatment records from 427 patients in the MIMIC-IV database were included in the study. Numerical features were compared using a two-sample t-test, while categorical features were compared using a chi-square test. A two-sided p-value less than 0.05 was considered statistically significant. The baseline characteristics of the selected patient and the treatment records can be found in Table 3. Among the records, 393 were successful treatments (78.9%) and 105 were failed treatments (21.1%). Table 3 highlights the presence of significant differences in terms of age, gender, BMI, duration of HFNC treatment, type of ICU, and mortality rate. In particular, the mortality rate for successful treatment records is significantly lower than that for failed treatment records by 24%.

**Table 3:** Baseline characteristics of patient treatment records. Plus–minus values are means ± SD.

	Overall	HFNC treatment success	HFNC treatment failure	P value
Age (year)	66.8 ± 14.9	67.0 ± 14.8	66.2 ± 15.4	< 0.05
Gender, n (% Men)	284(57.0)	222(56.5)	62(59.0)	< 0.05
BMI (kg/m^2^)	28.5 ± 7.8	28.8 ± 8.1	27.0 ± 6.3	< 0.05
Duration of HFNC treatment (h)	24.9 ± 10.2	25.3 ± 10.1	23.5 ± 10.3	< 0.05
Type of ICU, n (%)				< 0.05
CCU	47(9.4)	40(10.2)	7(6.7)	
SICU	51(10.2)	35(8.9)	16(15.2)	
NI	7(1.4)	6(1.5)	1(1.0)	
SICU	23(4.6)	17(4.3)	6(5.7)	
CVICU	58(11.6)	50(12.7)	8(7.6)	
TSICU	65(13.1)	49(12.5)	16(15.2)	
MICU	123(24.7)	98(24.9)	25(23.8)	
MICU/SICU	124(24.9)	98(24.9)	26(24.8)	
Mortality, n (%)	105(21.1)	63(16.0)	42(40.0)	< 0.05

### Performance in failure of HFNC treatment prediction

Table 4 presents the performance metrics for predicting HFNC treatment failure using five machine learning algorithms, one deep learning approach based on LSTM, and a soft voting ensemble method. The evaluation was conducted using a 1-h observation window and a 4-h prediction window.

**Table 4:** Machine learning indicators and 95% CI based on all features and common features, as well as traditional metrics, under the combination of a 1-h observation window and a 4-h prediction window.

	Model	ACC	AUC	SEN	SPE	BER	MCC	F1-score	KAPPA
All features	LR	0.765 (0.028–0.068)	0.822 (0.756–0.878)	0.776 (0.676–0.867)	0.764 (0.749–0.781)	0.230 (0.184–0.280)	0.208 (0.165–0.250)	0.160 (0.125–0.195)	0.114 (0.085–0.144)
	NB	0.806 (0.790–0.820)	0.776 (0.721–0.827)	0.645 (0.534–0.750)	0.810 (0.795–0.825)	0.272 (0.220–0.329)	0.189 (0.139–0.237)	0.160 (0.122–0.198)	0.116 (0.081–0.151)
	SVM	0.686 (0.668–0.704)	0.713 (0.640–0.783)	0.658 (0.548–0.764)	0.687 (0.669–0.705)	0.328 (0.275–0.384)	0.123 (0.081–0.163)	0.108 (0.081–0.135)	0.058 (0.037–0.080)
	RF	0.746 (0.729–0.763)	0.813 (0.761–0.862)	0.697 (0.587–0.798)	0.747 (0.730–0.764)	0.278 (0.227–0.333)	0.169 (0.124–0.210)	0.137 (0.104–0.170)	0.089 (0.062–0.117)
	LGBM	0.784 (0.768–0.799)	0.824 (0.768–0.876)	0.763 (0.662–0.857)	0.784 (0.768–0.800)	0.226 (0.179–0.277)	0.217 (0.170–0.261)	0.169 (0.131–0.208)	0.124 (0.092–0.158)
	Ensemble model	0.796 (0.780–0.811)	0.839 (0.786–0.889)	0.763 (0.667–0.857)	0.797 (0.781–0.812)	0.220 (0.173–0.270)	0.227 (0.179–0.270)	0.177 (0.138–0.216)	0.133 (0.099–0.168)
	LSTM	0.710 (0.692–0.727)	0.799 (0.739–0.854)	0.763 (0.663–0.857)	0.708 (0.690–0.726)	0.264 (0.197–0.279)	0.171 (0.130–0.211)	0.132 (0.101–0.163)	0.083 (0.059–0.109)
Common features	LR	0.679 (0.662–0.697)	0.767 (0.704–0.825)	0.763 (0.662–0.857)	0.677 (0.659–0.695)	0.280 (0.232–0.332)	0.156 (0.116–0.193)	0.121 (0.093–0.149)	0.071 (0.050–0.093)
	NB	0.836 (0.821–0.850)	0.723 (0.660–0.782)	0.408 (0.299–0.519)	0.849 (0.835–0.862)	0.372 (0.316–0.427)	0.117 (0.067–0.169)	0.125 (0.086–0.167)	0.080 (0.044–0.120)
	SVM	0.583 (0.564–0.602)	0.652 (0.587–0.715)	0.553 (0.437–0.662)	0.584 (0.564–0.603)	0.432 (0.376–0.491)	0.046 (0.006–0.084)	0.071 (0.050–0.092)	0.018 (0.002–0.033)
	RF	0.547 (0.528–0.566)	0.693 (0.627–0.758)	0.671 (0.562–0.778)	0.543 (0.523–0.563)	0.393 (0.339–0.448)	0.072 (0.035–0.108)	0.079 (0.060–0.099)	0.026 (0.012–0.040)
	LGBM	0.660 (0.642–0.679)	0.686 (0.620–0.750)	0.645 (0.536–0.755)	0.661 (0.643–0.680)	0.347 (0.292–0.403)	0.107 (0.066–0.148)	0.099 (0.074–0.124)	0.048 (0.028–0.068)
	Ensemble model	0.633 (0.615–0.651)	0.705 (0.637–0.767)	0.671 (0.562–0.772)	0.632 (0.613–0.650)	0.349 (0.298–0.404)	0.105 (0.065–0.141)	0.095 (0.071–0.120)	0.044 (0.026–0.062)
	LSTM	0.688 (0.670–0.705)	0.685 (0.612–0.753)	0.579 (0.463–0.691)	0.691 (0.673–0.709)	0.365 (0.309–0.424)	0.097 (0.054–0.138)	0.096 (0.070–0.123)	0.046 (0.025–0.068)
Traditional metrics	ROX	0.795 (0.761–0.829)	0.626 (0.580–0.673)	0.333 (0.243–0.424)	0.919 (0.891–0.946)	0.374 (0.327–0.421)	0.301 (0.195–0.404)	0.407 (0.312–0.497)	0.290 (0.188–0.392)
	mROX	0.562 (0.518–0.606)	0.559 (0.504–0.613)	0.552 (0.455–0.650)	0.565 (0.517–0.614)	0.441 (0.387–0.496)	0.096 (0.007–0.185)	0.347 (0.282–0.410)	0.082 (0.006–0.159)

**Fig. 5:**
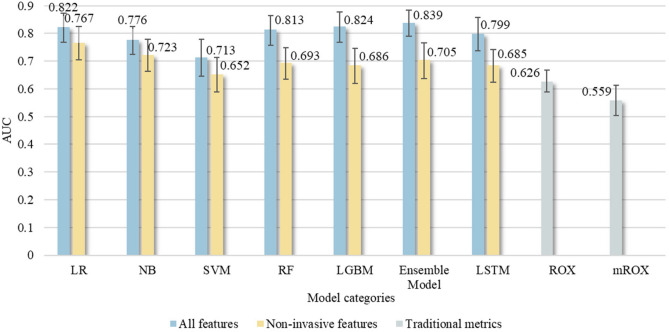
Comparison of AUC values for five machine learning methods, one deep learning method based on LSTM, and a soft voting ensemble algorithm using all features and only common features, along with the ROX and mROX indices. The error bars represent 95% confidence intervals. The observation and prediction windows are 1 h and 4 h, respectively.

Figure 5 presents the comparison of AUC values for five machine learning methods, a deep learning method based on LSTM, and the soft voting ensemble machine learning algorithm, using all features and only common features, as well as comparisons with the ROX and mROX indices. The results from Table 4 and Fig. 5 demonstrate the following: the soft voting ensemble machine learning algorithm achieved the highest AUC value of 0.839 (95% CI 0.786–0.889), when using all features. Correspondingly, among the individual machine learning methods, the LGBM model attained the highest AUC value of 0.824 (95% CI 0.768–0.876). When using only common features, LR yielded the highest AUC value of 0.767 (95% CI 0.704–0.825). Comparing the optimal group using only common features (based on LR) with the optimal group using all features (based on the ensemble model), there was a 0.072 difference in AUC values. All machine learning models outperformed the ROX index that had an AUC of 0.626 (95% CI 0.580–0.673), and the mROX index that had an AUC of 0.559 (95% CI 0.504–0.613). The ensemble model’s AUC was 0.839 on the original dataset and 0.811 in cross-database testing, decreasing by just 0.028. Similarly, the predictive performance of other machine learning and deep learning models declined somewhat after temporal validation (see Appendix 1 (Supplementary Table 2)). Despite this performance drop, the model outperformed traditional indices. This shows machine learning and deep learning algorithms are robust for disease prediction.

### Performance under different observation windows using only common features

**Fig. 6:**
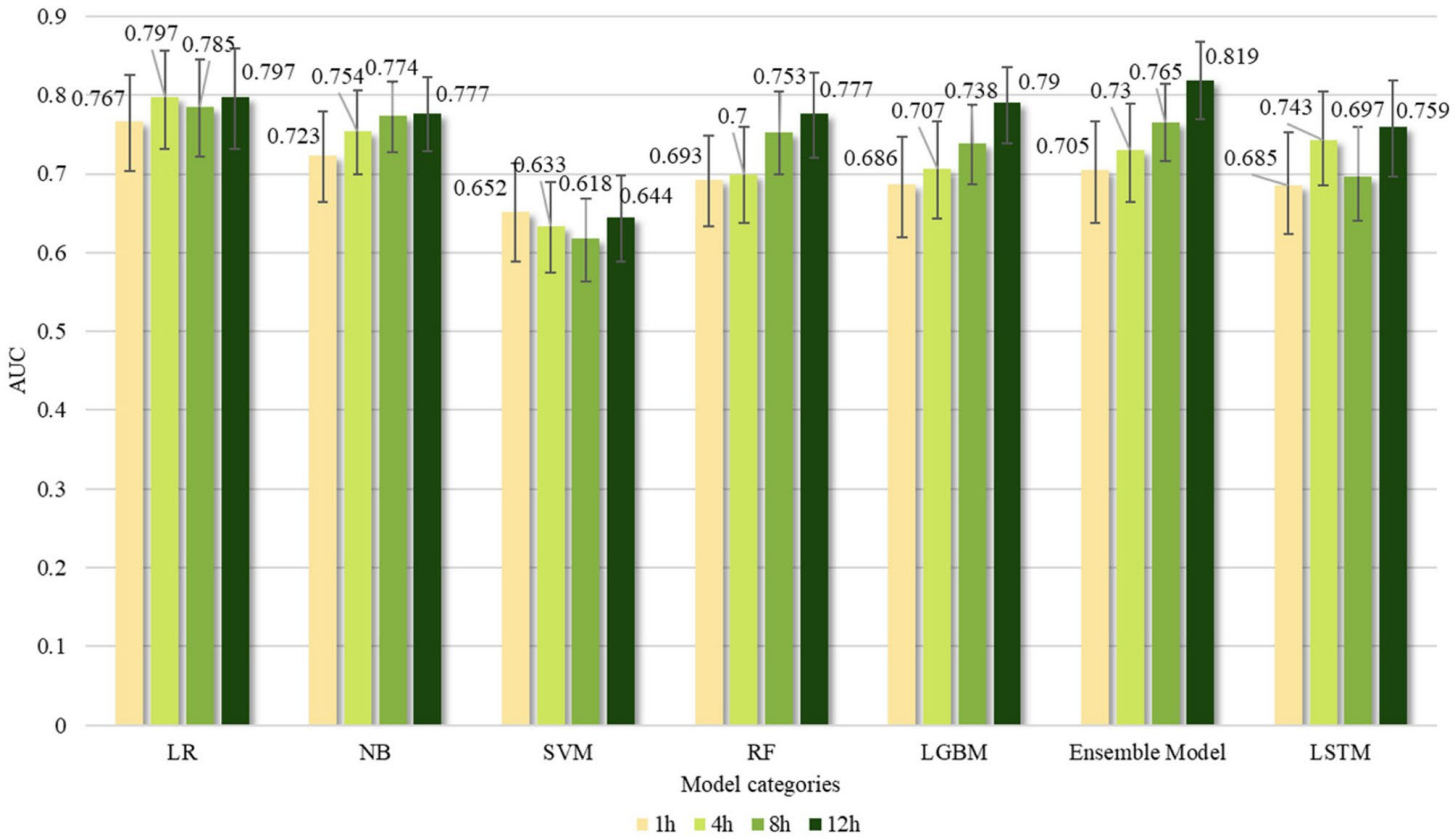
AUC values based on combinations of common features and multiple observation windows. The prediction window was standardized to a fixed duration of 4 h. The set observation window time (h) options included 1, 4, 8, and 12. The error bar represents 95%.

Figure 6 depicts the effect of HFNC treatment failure prediction using only common features under different observation windows, including 1, 4, 8, and 12 h with a 4-h prediction window. Under longer observation window settings, the AUC values show an overall increasing trend as the observation window time increased. Comparing the shortest 1-h observation window with the longest 12-h observation window, the LR model exhibited the lowest increase in AUC value at 0.03, while the soft voting ensemble machine learning algorithm demonstrated the highest increase at 0.114. The complete performance indicators for all window combinations can be found in Appendix 1 (Supplementary Table 1).

### Model interpretability analysis

**Fig. 7:**
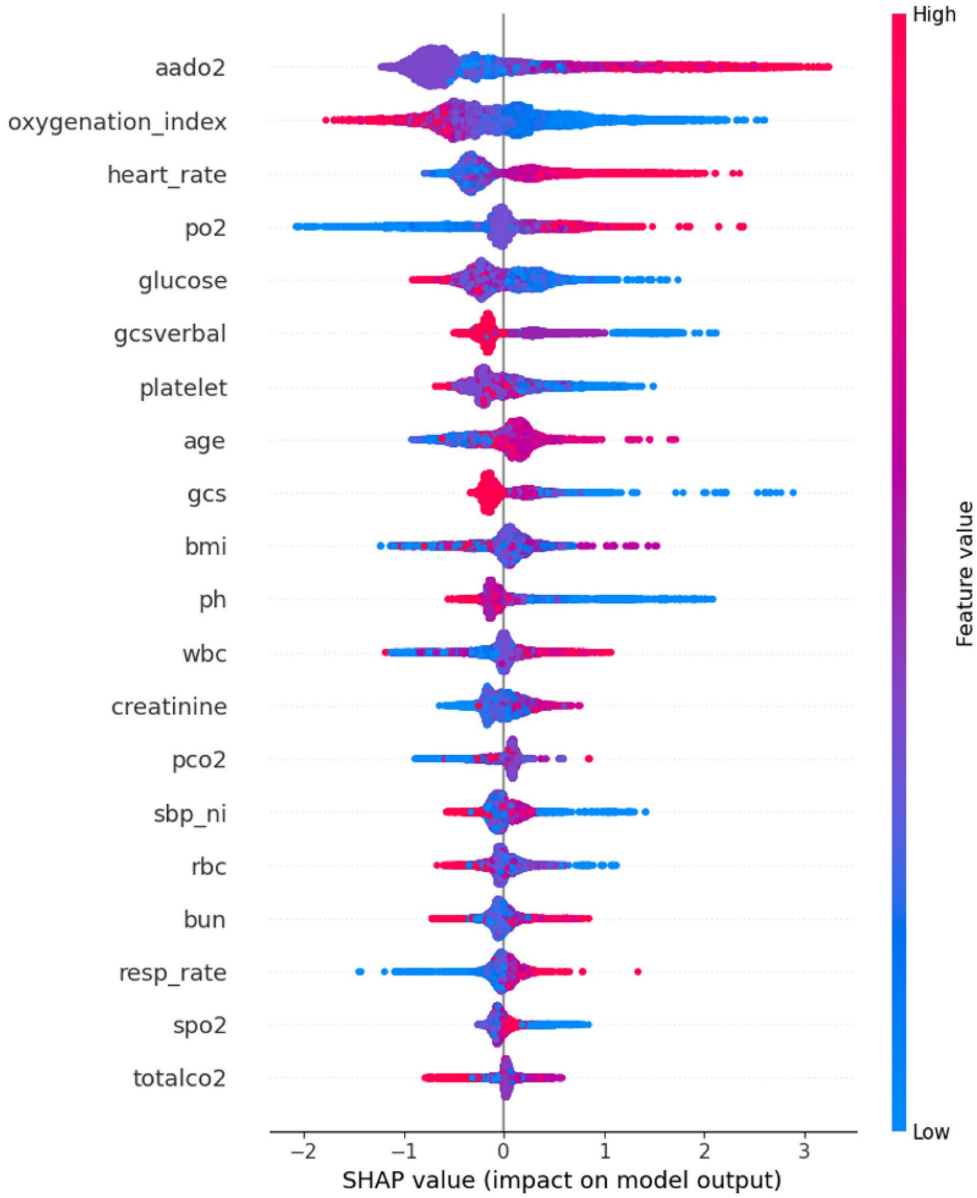
Interpretability analysis based on the SHAP Algorithm. The impact of eigenvalues on model predictions is illustrated. The left side of the figure displays the names of the features. The color bar on the right side represents the magnitude of the features, with red indicating high values and blue indicating low values. The horizontal axis corresponds to SHAP values. The density of the scatter plot signifies the distribution of the samples.

Interpretability analysis was performed using the SHAP algorithm with a 1-h observation window and a 4-h prediction window. Figure 7 shows the impact of the 20 most important features on the model’s predictions.

Figure 7 shows that the alveolar-arterial oxygen pressure difference (A-ado2) and heart rate were the laboratory and common features with the highest contributions to the model predictions, respectively. Taking A-ado2 as an example, when the SHAP value was positive, it was accompanied by high values of A-ado2. This means that as the A-ado2 value increases, the model tends to predict HFNC treatment failure. The oxygenation index (P/F) and arterial oxygen pressure (PO2) contributed less to the model predictions compared to A-ado2, but all three can be obtained through blood gas analysis. Therefore, subsequent research will focus on the investigation of the impact on model performance by incorporating three laboratory features into the non-invasive feature set through ablation experiments.

**Fig. 8:**
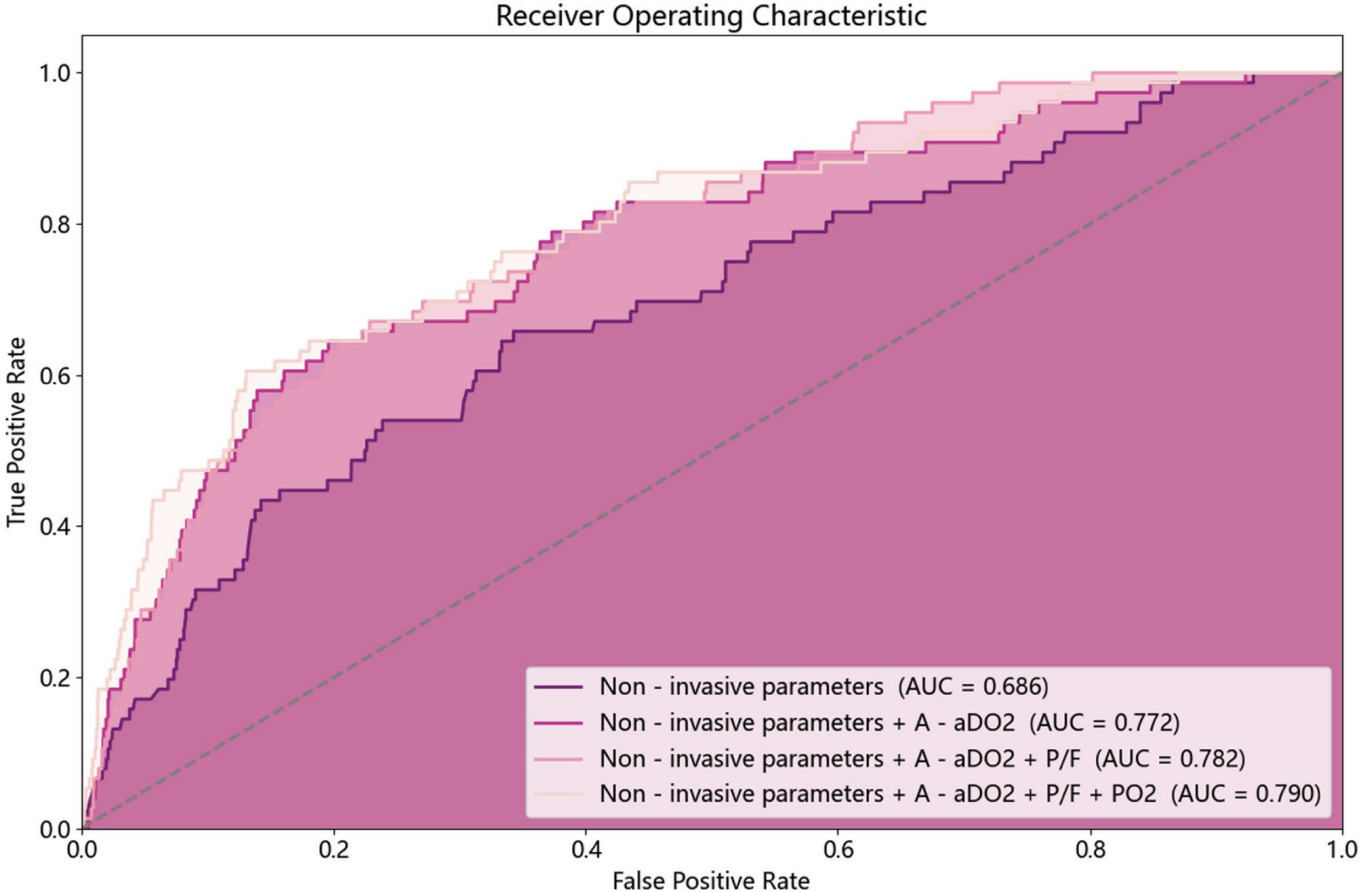
Comparing the AUC values of models using only common features with those from three ablation experiments (based on the LGBM model for instance). The predictive performance of the model gradually improved as blood gas analysis features increased.

Figure 8 shows the results of the prediction after conducting ablation experiments with a 1-h observation window and a 4-h prediction window. The addition of blood gas features significantly improved the predictive performance of the model using only non-invasive parameters. In particular, the inclusion of A-ado2 resulted in an 0.086 increase in AUC value. Furthermore, the addition of both A-ado2 and P/F led to a 0.096 improvement in AUC value. Incorporation of all three blood gas features led to a model performance increase by 0.104, with only a 0.034 difference in AUC value compared to the model using all features.

**Fig. 9:**
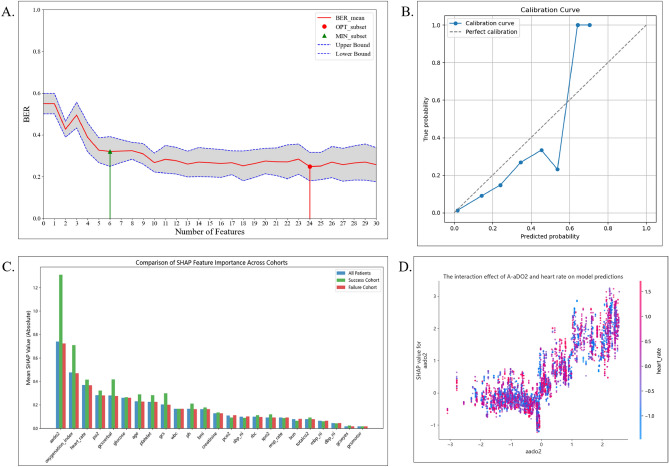
Interpretability analyses using RFECV, Brier score, and SHAP. (**A**) Recursive feature elimination with cross-validation (RFECV) was employed to balance model complexity and performance. A minimal subset of six features achieved near-optimal performance, while the full set of 24 features yielded the best cross-validation performance, albeit with substantially increased model complexity. (**B**) Calibration comparison (Brier scores) for different models under varied parameter settings; the ensemble model shows the lowest Brier scores, indicating superior calibration. (**C**) SHAP summary bar plot of mean absolute SHAP values for all patients (“All”), HFNC successes (“Success”), and failures (“Failure”); A-ado2 ranks highest across cohorts, followed by the P/F ratio. (**D**) SHAP interaction dependence plot for A-ado2 and heart rate: A-ado2 exhibits a nonlinear, positive contribution to failure risk, which is accentuated at higher heart rates.

To evaluate the trade-off between model complexity and predictive performance, we employed recursive feature elimination with cross-validation (RFECV), as shown in Fig. 9.

Figure 9 illustrates interpretability analyses using RFECV, Brier score, and SHAP. Specifically, panel A shows that a minimal subset of 6 features achieved strong predictive performance while reducing model complexity, it resulted in the lowest cross-validation error and the highest overall performance. This feature reduction strategy provided important guidance for selecting the final feature set and helped improve both the interpretability and predictive performance of the model.

Calibration was assessed using the Brier score across different models and parameter settings. As shown in Fig. 9, panel B, the ensemble model demonstrated lower Brier scores in most cases, indicating better overall calibration.

Figure 9, panel C shows a SHAP summary bar plot where A-ado2 had the highest mean SHAP value across all cohorts especially in successful HFNC cases followed by the P/F ratio.

As illustrated in Fig. 9, panel D, the SHAP interaction plot revealed a nonlinear effect of A-ado2 on HFNC failure risk, especially when coupled with tachycardia. These findings underscore the combined importance of gas-exchange impairment and compensatory physiological stress in predicting HFNC treatment escalation.

## Discussion

This study delved into the predictive performance of various machine learning methods, including the LSTM model, for HFNC treatment failure. We gauged model performance from multiple angles, including AUC and other classification metrics.

First, the results reveal that using only the common features yielded relatively good predictive results. As expected, the performance of the model using all features was superior to that of the model using only common features. However, invasive features are not easily obtainable in many primary healthcare facilities and pre-hospital environments. Compared to laboratory features, common features are easy to obtain from ordinary monitors and manual measurements, enhancing their application possibility. Using LR as an example, with a 1-h observation window, only a small difference in predictive performance between using only common features and using all features was present, namely a 0.055 difference in AUC value, which significantly outperformed the traditional ROX and mROX indices by 0.141 and 0.208, respectively. Therefore, when invasive monitoring is not feasible, non-invasive models can serve as potential auxiliary decision-making tools for HFNC treatment failure prediction in patients with AHRF.

Furthermore, increasing the width of the observation window had a positive impact on model performance. The predictive performance of using only common features showed an upward trend as the observation window duration increases. In case of the soft voting ensemble machine learning algorithm, the AUC value based on a 12-h observation window was 0.114 higher than that based on a 1-h observation window. This phenomenon indicates that when using only common features, a longer observation window captures more information, leading to more accurate predictions.

Additionally, an interpretability analysis was performed to analyze the feature weights. The interpretability analysis revealed that A-ado2, P/F, and PO2 ranked higher in their contributions to the model predictions among the laboratory features. An increase of A-ado2 typically indicates the presence of a diffusion impairment or ventilation/perfusion mismatch in the lungs, which can lead to inadequate oxygenation and deterioration of lung function. The high contribution degrees of P/F and PO2 indicate that even with high-flow and high-concentration inhaled oxygen, the patient cannot maintain normal oxygenation, suggesting the possibility of HFNC (high-flow nasal cannula) treatment failure and the need for timely adjustment of the treatment strategy. Ablation experiments confirmed that the performance of the non-invasive feature model improved by 0.104 after incorporating the three laboratory features of A-ado2, P/F, and PO2. Therefore, given their availability, the inclusion of high-contributing laboratory features will result in more reliable and accurate HFNC treatment failure predictions in patients with AHRF.

**Fig. 10:**
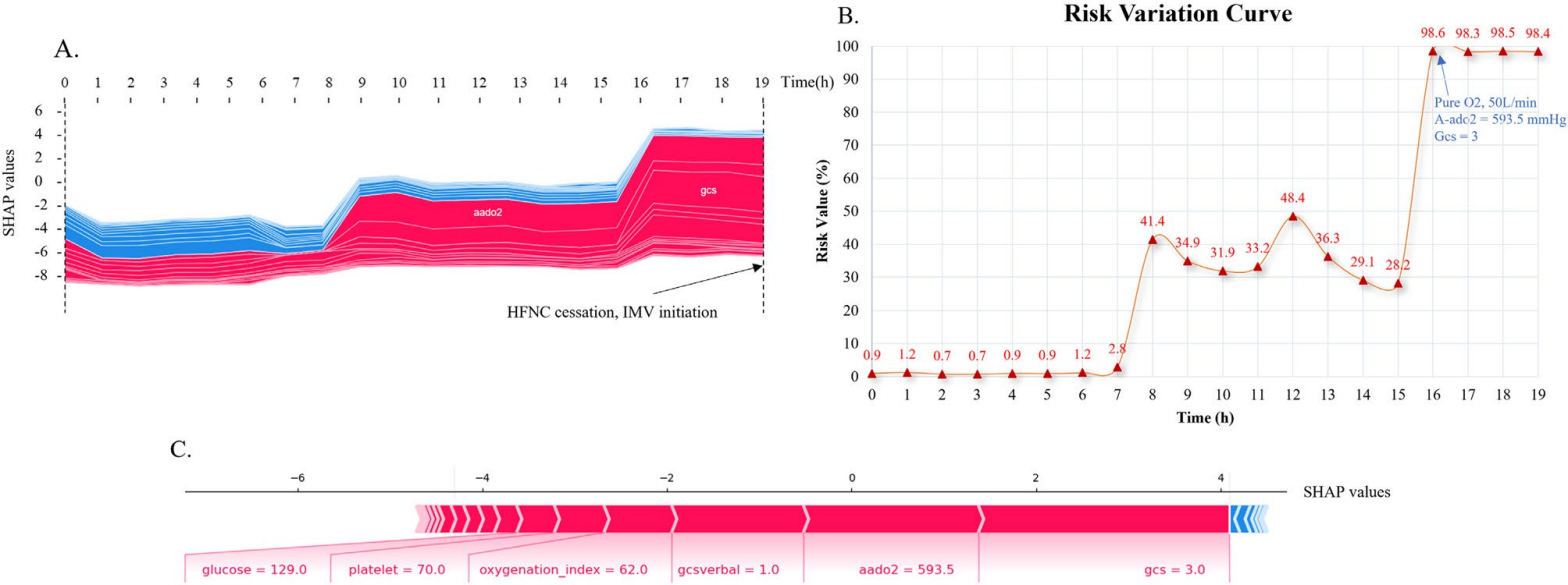
Analysis of a typical patient. The impact of features on prediction during HFNC treatment for a specific patient (10E). The x-axis represents the duration of HFNC treatment, while the y-axis indicates the degree of influence (SHAP value) of features on prediction. The risk values of treatment failure per hour during HFNC treatment (10F). The sharp value of features on prediction during the last hour of HFNC treatment (10G).

The real-time dynamic impact of typical patients’ physiological characteristics on model decisions was further investigated. Figure 10 presents an example analysis of a patient’s physiological characteristics and their influence on the model’s decisions. This was a 70-year-old Caucasian male who was admitted to the Medical/Surgical Intensive Care Unit (MICU/SICU) due to AHRF. The patient was placed on HFNC within 1 h of being admitted to the ward, upgraded to IMV after 19.5 h of treatment, and died after 136.5 h of IMV treatment.

As shown in Fig. 10, panel E, the proportion of high-risk features (in red) increased significantly after 8 h of HFNC treatment. After 16 h, the red area dominated, indicating a higher inclination of physiological characteristics towards predicting HFNC treatment failure. Figure 10, panel F illustrates the predicted hourly risk values of HFNC treatment failure throughout the therapy duration. Before the 8th hour of treatment, the risk value of HFNC treatment failure was low, indicating a low possibility of HFNC treatment failure. However, after 16 h of HFNC treatment, the total Glasgow Coma Scale score dropped to 3. The patient entered a state of coma, which lasted for 3.5 h before transitioning to IMV. The model predicted a risk value of HFNC treatment failure exceeding 98% at this point, consistent with Fig. 10E. Initiation of IMV therapy was consequently imperative. Panel G of Fig. 10 depicts the impact of physiological characteristics on predictions during the 19th hour of HFNC treatment. At this point, 25 features contribute to the model’s prediction of HFNC treatment failure.

In future applications, the proposed model could be integrated into patient monitoring devices or high-flow therapy equipment as an auxiliary tool for real-time alert and risk identification. For instance, in critical care settings, the algorithm could monitor dynamic changes in key physiological parameters to detect the risk of HFNC treatment failure in real time. This real-time alert system would provide clinical staff with vital decision support, enabling timely interventions that reduce the risk of delayed intubation and improve patient outcomes.

This study has several limitations. First, this is a single-center retrospective study based solely on the MIMIC-IV database. While the model showed good performance through internal temporal validation, further evaluation in larger, multi-center, and prospective clinical cohorts is necessary to establish its generalizability and robustness across diverse clinical environments. In future work, we plan to further explore and optimize models based solely on common features, particularly in real-time, prospective, and multi-institutional settings. Second, this study falls under the category of retrospective cohort research and has not been implemented in clinical practice, thus requiring further exploration of its clinical usability. Thirdly, we were only able to confirm the presence of acute hypoxemic respiratory failure (AHRF) in the patients. Although a subset of these patients likely had ARDS, the study lacks specific exploration of more prototypical ARDS cases, which limits the applicability of our findings to the broader ARDS population. Fourthly, although the present study was conducted using ICU data, the model was intentionally restricted to non-invasive inputs with the aim of simulating deployment in pre-hospital or resource-constrained environments. Analysis showed that 313 out of 427 patients (73.3%) received ABGA testing at the start of HFNC therapy, indicating such tests were typically part of routine baseline assessment rather than triggered by clinical deterioration. Nonetheless, some tests may still reflect clinician suspicion, potentially introducing subtle bias*.* Finally, while our model demonstrated strong predictive performance in retrospective data, real-world utility remains uncertain. Prior evidence suggests that models may perform significantly worse prospectively due to data missingness and workflow variability.

For example, the Rothman Index achieved AUROC 0.93 retrospectively but dropped to 0.73 in prospective validation, with no significant advantage over clinician judgment (Arnold et al., 2019). Similarly, the Epic sepsis model showed reduced sensitivity after deployment (Wong et al., 2021; Shimabukuro et al., 2023). These discrepancies highlight the well-recognized performance gap between retrospective development and prospective implementation. Therefore, prospective, multicenter validation is necessary to confirm clinical effectiveness and generalizability.

## Conclusion

In this study, a real-time dynamic alert model was developed to predict high-flow nasal cannula (HFNC) treatment failure in patients with acute hypoxemic respiratory failure (AHRF). The model incorporated five classical machine learning algorithms, a long short-term memory (LSTM) neural network for sequential data learning, and a soft voting ensemble algorithm to improve overall predictive performance. The results demonstrate that machine learning methods exhibit superior predictive performance compared to traditional prediction methods, such as the ROX and mROX indices. Additionally, the machine learning model shows considerably potential in HFNC treatment failure prediction using only common features. Therefore, this approach has potential to assist early risk identification of HFNC treatment failure, particularly in resource-limited environments. While the model shows encouraging internal performance, prospective validation and real-world testing are needed before clinical implementation.

## Supplementary Information

Supplementary Information.

## Data Availability

The data supporting the findings of this study are publicly available from the MIMIC-IV database (version 2.2) at https://physionet.org/content/mimiciv/2.2/. The dataset and source code used in this study are available from the corresponding author upon reasonable request.
