# Supplementary material for: Real-time prediction of HFNC treatment failure in acute hypoxemic respiratory failure using machine learning
Source: Sci Rep. 2025 Aug 18;15:30245. doi: 10.1038/s41598-025-16061-x (PMC12361419; doi:10.1038/s41598-025-16061-x)
Supplement: Supplementary file 1 — Supplementary Information. [file 41598_2025_16061_MOESM1_ESM.docx]

**Supplementary Table 1:**

(1) Machine learning indicators based on all features and common features, as well as traditional metrics, under the combination of an observation window of 1 hour and a prediction window of 4 hours.

|  | Model | ACC | AUC | SEN | SPE | BER | MCC | F1-score | KAPPA |
| --- | --- | --- | --- | --- | --- | --- | --- | --- | --- |
| all features | LR | 0.765  (0.028-0.068) | 0.822  (0.756-0.878) | 0.776  (0.676-0.867) | 0.764  (0.749-0.781) | 0.230  (0.184-0.280) | 0.208  (0.165-0.250) | 0.160  (0.125-0.195) | 0.114  (0.085-0.144) |
|  | NB | 0.806  (0.790-0.820) | 0.776  (0.721-0.827) | 0.645  (0.534-0.750) | 0.810  (0.795-0.825) | 0.272  (0.220-0.329) | 0.189  (0.139-0.237) | 0.160  (0.122-0.198) | 0.116  (0.081-0.151) |
|  | SVM | 0.686  (0.668-0.704) | 0.713  (0.640-0.783) | 0.658  (0.548-0.764) | 0.687  (0.669-0.705) | 0.328  (0.275-0.384) | 0.123  (0.081-0.163) | 0.108  (0.081-0.135) | 0.058  (0.037-0.080) |
|  | RF | 0.746  (0.729-0.763) | 0.813  (0.761-0.862) | 0.697  (0.587-0.798) | 0.747  (0.730-0.764) | 0.278  (0.227-0.333) | 0.169  (0.124-0.210) | 0.137  (0.104-0.170) | 0.089  (0.062-0.117) |
|  | LGBM | 0.784  (0.768-0.799) | 0.824  (0.768-0.876) | 0.763  (0.662-0.857) | 0.784  (0.768-0.800) | 0.226  (0.179-0.277) | 0.217  (0.170-0.261) | 0.169  (0.131-0.208) | 0.124  (0.092-0.158) |
|  | Ensemble Model | 0.796  (0.780-0.811) | 0.839  (0.786-0.889) | 0.763  (0.667-0.857) | 0.797  (0.781-0.812) | 0.220  (0.173-0.270) | 0.227  (0.179-0.270) | 0.177  (0.138-0.216) | 0.133  (0.099-0.168) |
|  | LSTM | 0.710  (0.692-0.727) | 0.799  (0.739-0.854) | 0.763  (0.663-0.857) | 0.708  (0.690-0.726) | 0.264  (0.197-0.279) | 0.171  (0.130-0.211) | 0.132  (0.101-0.163) | 0.083  (0.059-0.109) |
| common features | LR | 0.679  (0.662-0.697) | 0.767  (0.704-0.825) | 0.763  (0.662-0.857) | 0.677  (0.659-0.695) | 0.280  (0.232-0.332) | 0.156  (0.116-0.193) | 0.121  (0.093-0.149) | 0.071  (0.050-0.093) |
|  | NB | 0.836  (0.821-0.850) | 0.723  (0.660-0.782) | 0.408  (0.299-0.519) | 0.849  (0.835-0.862) | 0.372  (0.316-0.427) | 0.117  (0.067-0.169) | 0.125  (0.086-0.167) | 0.080  (0.044-0.120) |
|  | SVM | 0.583  (0.564-0.602) | 0.652  (0.587-0.715) | 0.553  (0.437-0.662) | 0.584  (0.564-0.603) | 0.432  (0.376-0.491) | 0.046  (0.006-0.084) | 0.071  (0.050-0.092) | 0.018  (0.002-0.033) |
|  | RF | 0.547  (0.528-0.566) | 0.693  (0.627-0.758) | 0.671  (0.562-0.778) | 0.543  (0.523-0.563) | 0.393  (0.339-0.448) | 0.072  (0.035-0.108) | 0.079  (0.060-0.099) | 0.026  (0.012-0.040) |
|  | LGBM | 0.660  (0.642-0.679) | 0.686  (0.620-0.750) | 0.645  (0.536-0.755) | 0.661  (0.643-0.680) | 0.347  (0.292-0.403) | 0.107  (0.066-0.148) | 0.099  (0.074-0.124) | 0.048  (0.028-0.068) |
|  | Ensemble Model | 0.633  (0.615-0.651) | 0.705  (0.637-0.767) | 0.671  (0.562-0.772) | 0.632  (0.613-0.650) | 0.349  (0.298-0.404) | 0.105  (0.065-0.141) | 0.095  (0.071-0.120) | 0.044  (0.026-0.062) |
|  | LSTM | 0.688  (0.670-0.705) | 0.685  (0.612-0.753) | 0.579  (0.463-0.691) | 0.691  (0.673-0.709) | 0.365  (0.309-0.424) | 0.097  (0.054-0.138) | 0.096  (0.070-0.123) | 0.046  (0.025-0.068) |
| traditional metrics | ROX | 0.795  (0.761-0.829) | 0.626  (0.580-0.673) | 0.333  (0.243-0.424) | 0.919  (0.891-0.946) | 0.374  (0.327-0.421) | 0.301  (0.195-0.404) | 0.407  (0.312-0.497) | 0.290  (0.188-0.392) |
|  | mROX | 0.562  (0.518-0.606) | 0.559  (0.504-0.613) | 0.552  (0.455-0.650) | 0.565  (0.517-0.614) | 0.441  (0.387-0.496) | 0.096  (0.007-0.185) | 0.347  (0.282-0.410) | 0.082  (0.006-0.159) |

**Supplementary Table 1:**

(2) Machine learning indicators based on all features and common features, as well as traditional metrics, under the combination of an observation window of 4 hour and a prediction window of 4 hours.

|  | Model | ACC | AUC | SEN | SPE | BER | MCC | F1-score | KAPPA |
| --- | --- | --- | --- | --- | --- | --- | --- | --- | --- |
| all features | LR | 0.772  (0.755-0.788) | 0.829  (0.769-0.881) | 0.816  (0.723-0.897) | 0.770  (0.753-0.787) | 0.207  (0.166-0.255) | 0.240  (0.194-0.283) | 0.188  (0.149-0.229) | 0.139  (0.105-0.174) |
|  | NB | 0.790  (0.773-0.806) | 0.761  (0.712-0.807) | 0.579  (0.468-0.690) | 0.797  (0.780-0.813) | 0.312  (0.256-0.369) | 0.162  (0.111-0.213) | 0.152  (0.112-0.192) | 0.101  (0.066-0.138) |
|  | SVM | 0.671  (0.652-0.689) | 0.746  (0.685-0.804) | 0.697  (0.595-0.798) | 0.670  (0.650-0.689) | 0.316  (0.265-0.368) | 0.137  (0.096-0.177) | 0.121  (0.092-0.151) | 0.066  (0.043-0.089) |
|  | RF | 0.694  (0.675-0.713) | 0.806  (0.754-0.855) | 0.750  (0.648-0.845) | 0.692  (0.672-0.712) | 0.279  (0.231-0.330) | 0.168  (0.125-0.208) | 0.138  (0.106-0.169) | 0.083  (0.058-0.109) |
|  | LGBM | 0.775  (0.758-0.792) | 0.830  (0.775-0.880) | 0.737  (0.631-0.833) | 0.776  (0.759-0.793) | 0.243  (0.195-0.298) | 0.213  (0.164-0.259) | 0.176  (0.136-0.216) | 0.125  (0.091-0.160) |
|  | Ensemble Model | 0.783  (0.766-0.799) | 0.839  (0.783-0.887) | 0.750  (0.649-0.847) | 0.784  (0.767-0.801) | 0.233  (0.185-0.284) | 0.224  (0.175-0.270) | 0.183  (0.142-0.224) | 0.134  (0.098-0.170) |
|  | LSTM | 0.722  (0.704-0.741) | 0.784  (0.730-0.835) | 0.724  (0.620-0.825) | 0.722  (0.704-0.741) | 0.277  (0.226-0.329) | 0.174  (0.130-0.218) | 0.145  (0.110-0.180) | 0.092  (0.048-0.121) |
| common features | LR | 0.766  (0.749-0.784) | 0.797  (0.736-0.852) | 0.737  (0.639-0.833) | 0.767  (0.750-0.785) | 0.248  (0.198-0.298) | 0.207  (0.162-0.252) | 0.170  (0.133-0.210) | 0.120  (0.088-0.153) |
|  | NB | 0.843  (0.828-0.858) | 0.754  (0.705-0.798) | 0.382  (0.276-0.493) | 0.859  (0.844-0.873) | 0.380  (0.323-0.433) | 0.120  (0.065-0.176) | 0.137  (0.094-0.181) | 0.088  (0.047-0.130) |
|  | SVM | 0.656  (0.636-0.675) | 0.633  (0.567-0.699) | 0.539  (0.429-0.651) | 0.660  (0.640-0.680) | 0.400  (0.343-0.457) | 0.074  (0.031-0.117) | 0.093  (0.067-0.120) | 0.035  (0.015-0.057) |
|  | RF | 0.678  (0.659-0.698) | 0.700  (0.637-0.761) | 0.645  (0.535-0.750) | 0.679  (0.660-0.699) | 0.338  (0.284-0.394) | 0.122  (0.079-0.164) | 0.115  (0.086-0.146) | 0.060  (0.037-0.083) |
|  | LGBM | 0.634  (0.614-0.654) | 0.707  (0.645-0.769) | 0.658  (0.548-0.767) | 0.633  (0.613-0.653) | 0.355  (0.299-0.410) | 0.107  (0.065-0.148) | 0.105  (0.078-0.132) | 0.048  (0.028-0.069) |
|  | Ensemble Model | 0.665  (0.646-0.683) | 0.730  (0.667-0.786) | 0.658  (0.547-0.762) | 0.665  (0.646-0.684) | 0.338  (0.286-0.395) | 0.121  (0.076-0.161) | 0.113  (0.085-0.142) | 0.057  (0.034-0.079) |
|  | LSTM | 0.657  (0.637-0.675) | 0.743  (0.683-0.799) | 0.724  (0.621-0.821) | 0.654  (0.634-0.673) | 0.311  (0.261-0.364) | 0.140  (0.098-0.179) | 0.120  (0.092-0.150) | 0.065  (0.043-0.087) |
| traditional metrics | ROX | 0.795  (0.761-0.829) | 0.626  (0.580-0.673) | 0.333  (0.243-0.424) | 0.919  (0.891-0.946) | 0.374  (0.327-0.421) | 0.301  (0.195-0.404) | 0.407  (0.312-0.497) | 0.290  (0.188-0.392) |
|  | mROX | 0.562  (0.518-0.606) | 0.559  (0.504-0.613) | 0.552  (0.455-0.650) | 0.565  (0.517-0.614) | 0.441  (0.387-0.496) | 0.096  (0.007-0.185) | 0.347  (0.282-0.410) | 0.082  (0.006-0.159) |

**Supplementary Table 1:**

(3) Machine learning indicators based on all features and common features, as well as traditional metrics, under the combination of an observation window of 8 hour and a prediction window of 4 hours.

|  | Model | ACC | AUC | SEN | SPE | BER | MCC | F1-score | KAPPA |
| --- | --- | --- | --- | --- | --- | --- | --- | --- | --- |
| all features | LR | 0.801  (0.783-0.819) | 0.832  (0.774-0.884) | 0.789  (0.693-0.880) | 0.801  (0.783-0.820) | 0.205  (0.160-0.253) | 0.276  (0.221-0.325) | 0.237  (0.188-0.285) | 0.183  (0.138-0.226) |
|  | NB | 0.787  (0.768-0.805) | 0.756  (0.708-0.802) | 0.605  (0.494-0.713) | 0.794  (0.776-0.812) | 0.300  (0.246-0.357) | 0.187  (0.132-0.240) | 0.182  (0.138-0.227) | 0.124  (0.083-0.165) |
|  | SVM | 0.609  (0.586-0.631) | 0.747  (0.687-0.801) | 0.684  (0.574-0.786) | 0.606  (0.583-0.628) | 0.355  (0.303-0.411) | 0.115  (0.069-0.157) | 0.121  (0.091-0.151) | 0.053  (0.031-0.076) |
|  | RF | 0.691  (0.670-0.712) | 0.775  (0.716-0.828) | 0.737  (0.634-0.835) | 0.689  (0.667-0.709) | 0.287  (0.237-0.340) | 0.176  (0.129-0.221) | 0.158  (0.122-0.194) | 0.094  (0.066-0.124) |
|  | LGBM | 0.735  (0.715-0.755) | 0.825  (0.772-0.876) | 0.750  (0.652-0.846) | 0.734  (0.714-0.755) | 0.258  (0.210-0.308) | 0.208  (0.160-0.254) | 0.182  (0.141-0.222) | 0.121  (0.087-0.156) |
|  | Ensemble Model | 0.746  (0.727-0.765) | 0.837  (0.785-0.884) | 0.750  (0.652-0.841) | 0.746  (0.726-0.765) | 0.252  (0.205-0.304) | 0.216  (0.167-0.263) | 0.188  (0.147-0.230) | 0.128  (0.094-0.165) |
|  | LSTM | 0.696  (0.675-0.716) | 0.772  (0.715-0.824) | 0.671  (0.557-0.778) | 0.697  (0.675-0.717) | 0.316  (0.262-0.373) | 0.154  (0.103-0.200) | 0.148  (0.112-0.184) | 0.084  (0.053-0.114) |
| common features | LR | 0.692  (0.672-0.712) | 0.785  (0.723-0.841) | 0.750  (0.649-0.843) | 0.690  (0.669-0.710) | 0.280  (0.232-0.332) | 0.182  (0.135-0.226) | 0.161  (0.124-0.197) | 0.097  (0.068-0.127) |
|  | NB | 0.822  (0.805-0.838) | 0.774  (0.733-0.813) | 0.434  (0.325-0.547) | 0.838  (0.821-0.854) | 0.364  (0.307-0.418) | 0.140  (0.082-0.198) | 0.161  (0.114-0.208) | 0.104  (0.059-0.150) |
|  | SVM | 0.581  (0.559-0.603) | 0.618  (0.551-0.681) | 0.526  (0.413-0.637) | 0.583  (0.561-0.605) | 0.445  (0.389-0.504) | 0.043  (-0.003-0.088) | 0.090  (0.064-0.116) | 0.019  (-0.001-0.041) |
|  | RF | 0.707  (0.687-0.728) | 0.753  (0.698-0.803) | 0.711  (0.606-0.811) | 0.707  (0.686-0.728) | 0.291  (0.240-0.346) | 0.176  (0.127-0.222) | 0.160  (0.123-0.199) | 0.097  (0.066-0.129) |
|  | LGBM | 0.679  (0.657-0.699) | 0.738  (0.684-0.791) | 0.697  (0.592-0.800) | 0.678  (0.657-0.699) | 0.312  (0.261-0.365) | 0.154  (0.107-0.199) | 0.145  (0.111-0.181) | 0.081  (0.054-0.109) |
|  | Ensemble Model | 0.710  (0.690-0.730) | 0.765  (0.711-0.815) | 0.671  (0.563-0.773) | 0.712  (0.691-0.732) | 0.309  (0.255-0.365) | 0.162  (0.111-0.209) | 0.154  (0.117-0.191) | 0.090  (0.060-0.122) |
|  | LSTM | 0.665  (0.644-0.686) | 0.697  (0.648-0.742) | 0.632  (0.524-0.740) | 0.666  (0.645-0.687) | 0.351  (0.296-0.407) | 0.122  (0.075-0.168) | 0.129  (0.097-0.162) | 0.063  (0.037-0.090) |
| traditional metrics | ROX | 0.795  (0.761-0.829) | 0.626  (0.580-0.673) | 0.333  (0.243-0.424) | 0.919  (0.891-0.946) | 0.374  (0.327-0.421) | 0.301  (0.195-0.404) | 0.407  (0.312-0.497) | 0.290  (0.188-0.392) |
|  | mROX | 0.562  (0.518-0.606) | 0.559  (0.504-0.613) | 0.552  (0.455-0.650) | 0.565  (0.517-0.614) | 0.441  (0.387-0.496) | 0.096  (0.007-0.185) | 0.347  (0.282-0.410) | 0.082  (0.006-0.159) |

**Supplementary Table 1:**

(4) Machine learning indicators based on all features and common features, as well as traditional metrics, under the combination of an observation window of 12 hour and a prediction window of 4 hours.

|  | Model | ACC | AUC | SEN | SPE | BER | MCC | F1-score | KAPPA |
| --- | --- | --- | --- | --- | --- | --- | --- | --- | --- |
| all features | LR | 0.777  (0.756-0.798) | 0.825  (0.766-0.880) | 0.829  (0.737-0.915) | 0.775  (0.753-0.796) | 0.198  (0.154-0.245) | 0.289  (0.234-0.342) | 0.253  (0.200-0.305) | 0.190  (0.144-0.236) |
|  | NB | 0.794  (0.773-0.814) | 0.748  (0.683-0.807) | 0.643  (0.525-0.758) | 0.801  (0.780-0.821) | 0.278  (0.219-0.337) | 0.224  (0.160-0.285) | 0.221  (0.167-0.276) | 0.158  (0.108-0.209) |
|  | SVM | 0.646  (0.621-0.669) | 0.704  (0.637-0.766) | 0.629  (0.513-0.735) | 0.646  (0.621-0.670) | 0.362  (0.307-0.422) | 0.119  (0.066-0.167) | 0.139  (0.103-0.175) | 0.063  (0.033-0.092) |
|  | RF | 0.693  (0.670-0.717) | 0.773  (0.718-0.827) | 0.714  (0.611-0.817) | 0.692  (0.668-0.716) | 0.297  (0.245-0.349) | 0.181  (0.130-0.231) | 0.175  (0.135-0.217) | 0.103  (0.071-0.139) |
|  | LGBM | 0.754  (0.733-0.776) | 0.831  (0.778-0.883) | 0.771  (0.671-0.869) | 0.753  (0.732-0.775) | 0.238  (0.188-0.289) | 0.246  (0.191-0.300) | 0.222  (0.173-0.273) | 0.156  (0.113-0.201) |
|  | Ensemble Model | 0.776  (0.755-0.796) | 0.836  (0.778-0.889) | 0.786  (0.689-0.879) | 0.775  (0.754-0.796) | 0.220  (0.172-0.268) | 0.270  (0.215-0.322) | 0.242  (0.191-0.292) | 0.178  (0.133-0.224) |
|  | LSTM | 0.656  (0.633-0.680) | 0.730  (0.665-0.791) | 0.657  (0.546-0.767) | 0.656  (0.632-0.681) | 0.343  (0.287-0.401) | 0.136  (0.084-0.187) | 0.148  (0.111-0.187) | 0.073  (0.043-0.104) |
| common features | LR | 0.714  (0.691-0.737) | 0.797  (0.732-0.854) | 0.771  (0.667-0.866) | 0.711  (0.688-0.735) | 0.259  (0.210-0.313) | 0.217  (0.162-0.268) | 0.197  (0.152-0.242) | 0.128  (0.091-0.167) |
|  | NB | 0.814  (0.794-0.834) | 0.777  (0.734-0.817) | 0.500  (0.382-0.618) | 0.829  (0.809-0.848) | 0.335  (0.276-0.396) | 0.176  (0.109-0.241) | 0.197  (0.142-0.252) | 0.133  (0.081-0.187) |
|  | SVM | 0.528  (0.503-0.552) | 0.644  (0.577-0.708) | 0.586  (0.470-0.700) | 0.525  (0.500-0.550) | 0.445  (0.386-0.504) | 0.046  (-0.003-0.094) | 0.101  (0.073-0.131) | 0.020  (-0.001-0.042) |
|  | RF | 0.709  (0.686-0.730) | 0.777  (0.716-0.830) | 0.743  (0.636-0.841) | 0.707  (0.684-0.730) | 0.275  (0.225-0.329) | 0.202  (0.147-0.253) | 0.188  (0.144-0.234) | 0.118  (0.081-0.157) |
|  | LGBM | 0.731  (0.709-0.753) | 0.790  (0.737-0.837) | 0.714  (0.603-0.820) | 0.732  (0.709-0.754) | 0.277  (0.223-0.333) | 0.206  (0.150-0.259) | 0.195  (0.150-0.239) | 0.126  (0.088-0.166) |
|  | Ensemble Model | 0.737  (0.715-0.758) | 0.819  (0.770-0.862) | 0.729  (0.623-0.831) | 0.737  (0.714-0.759) | 0.267  (0.214-0.323) | 0.215  (0.159-0.268) | 0.201  (0.155-0.249) | 0.133  (0.093-0.174) |
|  | LSTM | 0.691  (0.668-0.714) | 0.759  (0.714-0.801) | 0.657  (0.544-0.765) | 0.693  (0.670-0.716) | 0.325  (0.270-0.382) | 0.156  (0.104-0.207) | 0.162  (0.123-0.204) | 0.090  (0.057-0.125) |
| traditional metrics | ROX | ROX | 0.795  (0.761-0.829) | 0.626  (0.580-0.673) | 0.333  (0.243-0.424) | 0.919  (0.891-0.946) | 0.374  (0.327-0.421) | 0.301  (0.195-0.404) | 0.407  (0.312-0.497) |
|  | mROX | 0.562  (0.518-0.606) | 0.559  (0.504-0.613) | 0.552  (0.455-0.650) | 0.565  (0.517-0.614) | 0.441  (0.387-0.496) | 0.096  (0.007-0.185) | 0.347  (0.282-0.410) | 0.082  (0.006-0.159) |

**Supplementary Table 2:**

Based on a 1 hour observation window and a 4 hour prediction window, the entire model was validated across time using data from 2008 to 2013 as the training set and data from 2014 to 2019 as the testing set.

|  | Model | ACC | AUC | SEN | SPE | BER | MCC | F1-score | KAPPA |
| --- | --- | --- | --- | --- | --- | --- | --- | --- | --- |
| all features | LR | 0.670  (0.659-0.681) | 0.781  (0.751-0.814) | 0.737  (0.683-0.797) | 0.667  (0.656-0.679) | 0.298  (0.268-0.325) | 0.150  (0.128-0.174) | 0.126  (0.109-0.143) | 0.071  (0.059-0.085) |
|  | NB | 0.670  (0.659-0.681) | 0.748  (0.710-0.784) | 0.698  (0.636-0.757) | 0.669  (0.658-0.680) | 0.316  (0.287-0.348) | 0.137  (0.111-0.159) | 0.120  (0.103-0.136) | 0.065  (0.052-0.078) |
|  | SVM | 0.687  (0.676-0.697) | 0.774  (0.749-0.801) | 0.746  (0.692-0.805) | 0.685  (0.673-0.695) | 0.285  (0.254-0.312) | 0.162  (0.139-0.186) | 0.133  (0.116-0.151) | 0.079  (0.066-0.093) |
|  | RF | 0.715  (0.704-0.725) | 0.818  (0.792-0.843) | 0.746  (0.686-0.799) | 0.714  (0.703-0.724) | 0.270  (0.243-0.300) | 0.177  (0.151-0.200) | 0.144  (0.124-0.163) | 0.091  (0.075-0.107) |
|  | LGBM | 0.702  (0.691-0.712) | 0.758  (0.723-0.792) | 0.711  (0.651-0.769) | 0.701  (0.691-0.712) | 0.294  (0.264-0.324) | 0.157  (0.131-0.181) | 0.133  (0.114-0.151) | 0.079  (0.064-0.094) |
|  | Ensemble Model | 0.711  (0.700-0.721) | 0.811  (0.786-0.836) | 0.759  (0.702-0.812) | 0.709  (0.698-0.720) | 0.266  (0.239-0.295) | 0.179  (0.154-0.203) | 0.144  (0.124-0.163) | 0.092  (0.076-0.106) |
|  | LSTM | 0.744  (0.733-0.754) | 0.806  (0.778-0.836) | 0.759  (0.707-0.815) | 0.743  (0.733-0.753) | 0.249  (0.221-0.276) | 0.199  (0.175-0.224) | 0.160  (0.139-0.181) | 0.109  (0.092-0.127) |
| common features | LR | 0.626  (0.614-0.637) | 0.655  (0.617-0.696) | 0.595  (0.534-0.662) | 0.627  (0.615-0.638) | 0.389  (0.355-0.420) | 0.081  (0.058-0.106) | 0.093  (0.079-0.108) | 0.036  (0.025-0.048) |
|  | NB | 0.657  (0.646-0.668) | 0.703  (0.669-0.740) | 0.642  (0.586-0.707) | 0.658  (0.646-0.669) | 0.350  (0.317-0.379) | 0.111  (0.089-0.136) | 0.108  (0.093-0.124) | 0.052  (0.040-0.065) |
|  | SVM | 0.600  (0.589-0.611) | 0.599  (0.564-0.634) | 0.543  (0.483-0.609) | 0.602  (0.591-0.613) | 0.427  (0.395-0.458) | 0.052  (0.030-0.076) | 0.080  (0.068-0.094) | 0.022  (0.013-0.033) |
|  | RF | 0.661  (0.650-0.672) | 0.663  (0.629-0.701) | 0.573  (0.510-0.639) | 0.664  (0.653-0.675) | 0.381  (0.348-0.414) | 0.088  (0.064-0.114) | 0.098  (0.083-0.114) | 0.042  (0.030-0.055) |
|  | LGBM | 0.618  (0.607-0.629) | 0.644  (0.611-0.679) | 0.565  (0.502-0.630) | 0.620  (0.609-0.631) | 0.408  (0.374-0.439) | 0.067  (0.044-0.092) | 0.087  (0.073-0.101) | 0.029  (0.019-0.041) |
|  | Ensemble Model | 0.598 | 0.662 | 0.625 | 0.597 | 0.389 | 0.080 | 0.091 | 0.033 |
|  |  | (0.586-0.609) | (0.629-0.697) | (0.564-0.691) | (0.585-0.608) | (0.356-0.420) | (0.057-0.104) | (0.078-0.105) | (0.023-0.044) |
|  | LSTM | 0.616  (0.605-0.628) | 0.713  (0.680-0.747) | 0.677  (0.617-0.739) | 0.614  (0.603-0.626) | 0.355  (0.323-0.385) | 0.105  (0.082-0.129) | 0.102  (0.087-0.117) | 0.045  (0.034-0.056) |

**Supplementary Table 3:**

Missing rate analysis and exclusion.

| **index** | **feature names** | **% of total values** | **notes** | **reasons** |
| --- | --- | --- | --- | --- |
| 1 | wbc | 92.9 |  |  |
| 2 | rbc | 92.9 |  |  |
| 3 | platelet | 92.9 |  |  |
| 4 | hemoglobin | 92.9 | Not included in the analysis. | Excluded by the optimal feature subset algorithm. |
| 5 | hematocrit | 92.8 | Not included in the analysis. | Excluded by the optimal feature subset algorithm. |
| 6 | oxygenation_index | 92.6 |  |  |
| 7 | aado2 | 92.6 |  |  |
| 8 | baseexcess | 92.1 | Not included in the analysis. | Excluded by the optimal feature subset algorithm. |
| 9 | totalco2 | 92.1 |  |  |
| 10 | pco2 | 92.1 |  |  |
| 11 | ph | 92.1 |  |  |
| 12 | po2 | 92.1 |  |  |
| 13 | glucose | 90.9 |  |  |
| 14 | bun | 90.8 |  |  |
| 15 | creatinine | 90.8 |  |  |
| 16 | gcs_motor | 74.4 |  |  |
| 17 | gcs_verbal | 74.4 |  |  |
| 18 | gcs_eyes | 74.3 |  |  |
| 19 | gcs | 74.3 |  |  |
| 20 | temperature | 69.2 | Not included in the analysis. | Excluded by the optimal feature subset algorithm. |
| 21 | o2_flow | 60.9 | Not included in the analysis. | Excessive human intervention factors. |
| 22 | fio2 | 60.8 | Not included in the analysis. | Excessive human intervention factors. |
| 23 | urineoutput | 42.5 | Not included in the analysis. | Excluded by the optimal feature subset algorithm. |
| 24 | mbp_ni | 20.4 |  |  |
| 25 | sbp_ni | 20.4 |  |  |
| 26 | dbp_ni | 20.4 |  |  |
| 27 | spo2 | 0.8 |  |  |
| 28 | resp_rate | 0.7 |  |  |
| 29 | heart_rate | 0.7 |  |  |
| 30 | bmi | 0 |  |  |
| 31 | gender | 0 | Not included in the analysis. | Excluded by the optimal feature subset algorithm. |
| 32 | age | 0 |  |  |

**Supplementary Table 4:**

Model hyperparameter.

| Model | Parameter Name | Value |
| --- | --- | --- |
| LR | penalty | L1 |
|  | C | 0.5 |
|  | max_iter | 10000 |
|  | solver | liblinear |
| NB | priors | None |
|  | var_smoothing | $1e^{-9}$ |
|  | algorithm | auto |
|  | n_jobs | None |
| SVM | probability | True |
|  | class_weight | balanced |
|  | random_state | 30 |
| RF | n_estimators | 50 |
|  | max_depth | 10 |
|  | min_samples_split | 2 |
|  | min_samples_leaf | 4 |
|  | random_state | 90 |
| LGBM | boosting_type | gbdt |
|  | objective | binary |
|  | metric | binary_error |
|  | learning_rate | 0.05 |
|  | n_estimators | 100 |
|  | max_depth | 10 |
|  | num_leaves | 31 |
|  | subsample | 0.7 |
|  | reg_alpha | 0.01 |
|  | reg_lambda | 0.01 |
| LSTM | learning_rate | 0.001 |
|  | loss | binary_crossentropy |
|  | metrics | accuracy |
|  | units | 64 |
|  | rate | 0.3 |
|  | units | 32 |
|  | activation | sigmoid |
|  | epochs | 100 |
|  | batch_size | 32 |

**Supplementary Table 5:**

Brier Score.

|  | Model | 1-4 | 4-4 | 8-4 | 12-4 |
| --- | --- | --- | --- | --- | --- |
| all features | LR | 0.025  (0.020-0.031) | 0.029  (0.023-0.035) | 0.034  (0.027-0.040) | 0.040  (0.032-0.048) |
|  | NB | 0.082  (0.072-0.092) | 0.143  (0.129-0.157) | 0.181  (0.164-0.198) | 0.182  (0.163-0.201) |
|  | SVM | 0.027  (0.021-0.032) | 0.030  (0.024-0.037) | 0.037  (0.029-0.045) | 0.042  (0.033-0.051) |
|  | RF | 0.026  (0.021-0.031) | 0.028  (0.023-0.034) | 0.034  (0.028-0.041) | 0.039  (0.032-0.047) |
|  | LGBM | 0.033  (0.028-0.038) | 0.035  (0.029-0.041) | 0.040  (0.033-0.047) | 0.043  (0.034-0.051) |
|  | Ensemble Model | 0.026  (0.021-0.031) | 0.028  (0.023-0.034) | 0.033  (0.027-0.039) | 0.037  (0.030-0.045) |
|  | LSTM | 0.026  (0.021-0.031) | 0.031  (0.025-0.037) | 0.035  (0.028-0.042) | 0.043  (0.035-0.052) |
| common features | LR | 0.027  (0.022-0.033) | 0.030  (0.024-0.036) | 0.035  (0.028-0.042) | 0.040  (0.032-0.048) |
|  | NB | 0.092  (0.081-0.103) | 0.131  (0.118-0.144) | 0.144  (0.129-0.159) | 0.158  (0.140-0.176) |
|  | SVM | 0.028  (0.023-0.034) | 0.032  (0.026-0.039) | 0.040  (0.032-0.047) | 0.046  (0.038-0.055) |
|  | RF | 0.028  (0.023-0.034) | 0.031  (0.025-0.037) | 0.037  (0.030-0.044) | 0.041  (0.033-0.050) |
|  | LGBM | 0.046  (0.042-0.050) | 0.047  (0.043-0.052) | 0.050  (0.045-0.056) | 0.047  (0.041-0.054) |
|  | Ensemble Model | 0.032  (0.027-0.037) | 0.034  (0.029-0.040) | 0.039  (0.033-0.045) | 0.042  (0.034-0.049) |
|  | LSTM | 0.029  (0.023-0.034) | 0.031  (0.025-0.038) | 0.038  (0.030-0.045) | 0.042  (0.034-0.049) |

**Supplementary note:**

Regarding d) HFNC treatment duration between 12 and 48 hours：

This inclusion criterion was based on existing evidence and expert consensus. First, a meta-analysis by Zhen et al.^1^ reported that at 6 hours after HFNC initiation, the pooled sensitivity and specificity of the ROX index for predicting treatment failure were 0.67 (95% CI: 0.54–0.78) and 0.70 (95% CI: 0.60–0.78), respectively. However, the ROX index demonstrated better predictive performance when assessed at 12 hours, with a sensitivity of 0.73 (95% CI: 0.67–0.79) and specificity of 0.74 (95% CI: 0.70–0.78).

Second, in the “Expert Consensus on the Clinical Application of High-Flow Nasal Cannula Oxygen Therapy in Adults,” Xie et al.^2^ emphasized that a ROX index ≥4.88 at 12 hours post-HFNC initiation is a key indicator of treatment success for patients with acute type I respiratory failure ^3^. In clinical practice, it is generally believed that a minimum of 12 hours of HFNC therapy is required to effectively improve oxygenation, enhance patient comfort, and ensure compliance.

Regarding the upper limit of 48 hours, a study by Kang et al.^4^ in 2015 found that failure of HFNC beyond 48 hours was significantly associated with increased ICU mortality, lower extubation and weaning success rates, and fewer ventilator-free days. Specifically, patients who failed HFNC beyond 48 hours had significantly higher mortality than those who failed within 48 hours (66.7% vs. 39.2%; P = 0.001).

Therefore, in this study, we restricted the inclusion window for HFNC treatment duration to 12–48 hours. This decision balances the need to compare machine learning performance with the most validated ROX index timepoints, while ensuring that the analysis remains within a clinically safe and meaningful treatment window.

[1] JUNHAI Z, JING Y, BEIBEI C, et al. The value of ROX index in predicting the outcome of high flow nasal cannula: a systematic review and meta-analysis[J]. Respiratory Research, 2022, 23(1): 33.

[2] Xie L, Xu J, Yan P, et al. Expert consensus on the clinical application of high-flow nasal cannula oxygen therapy. Chinese Journal of Tuberculosis and Respiratory Diseases, 2019;42(2):83–91.

[3] ROCA O, MESSIKA J, CARALT B, et al. Predicting success of high-flow nasal cannula in pneumonia patients with hypoxemic respiratory failure: The utility of the ROX index[J]. Journal of Critical Care, 2016, 35: 200-205.

[4] KANG B J, KOH Y, LIM C M, et al. Failure of high-flow nasal cannula therapy may delay intubation and increase mortality[J]. Intensive Care Medicine, 2015, 41(4): 623-632.

**Supplementary Table 6:**

The classification, full names, and descriptions of all features included in the study.

|  | Category | Features | Explanation |
| --- | --- | --- | --- |
| common features | Demographic features | Age | Age at admission |
|  |  | BMI | BMI at admission |
|  |  | Gender | Gender of the patient |
|  | Physiological features | Urineoutput | Total hourly urine output of the patient |
|  |  | Heart rate | Patient's heart rate |
|  |  | Non-invasive systolic blood pressure | Patient's non-invasive systolic blood pressure |
|  |  | Non-invasive diastolic blood pressure | Patient's non-invasive diastolic blood pressure |
|  |  | Non-invasive mean blood pressure | Patient's non-invasive mean blood pressure |
|  |  | Respiration rate | Patient's respiratory rate |
|  |  | Temperature | Patient's temperature |
|  |  | SpO2 | Patient's oxygen saturation |
|  | Glasgow Scoring | Gcs | Glasgow Coma Scale total score |
|  |  | Gcsverbal | Glasgow Coma Scale verbal response score |
|  |  | Gcsmotor | Glasgow Coma Scale motor response score |
|  |  | Gcseyes | Glasgow Coma Scale eye opening response score |
| Laboratory features | Blood gas analysis features | Oxygenation_index | The ratio of arterial oxygen partial pressure to fractional inspired oxygen |
|  |  | A-ado2 | The alveolar-arterial oxygen gradient |
|  |  | Baseexcess | Base excess |
|  |  | TotalCO2 | Total carbon dioxide content |
|  |  | PCO2 | Arterial carbon dioxide partial pressure |
|  |  | PH | Blood pH |
|  |  | PO2 | Arterial oxygen partial pressure |
|  | Chemical assay features | Creatinine | Creatinine concentration |
|  |  | Glucose | Blood glucose level |
|  |  | Bun | Blood urea nitrogen concentration |
|  | Complete blood cell count features | Hematocrit | Percentage of blood volume occupied by red blood cells |
|  |  | Hemoglobin | Hemoglobin concentration in blood |
|  |  | Platelet | Platelet count in blood |
|  |  | Rbc | Red blood cell count in blood |
|  |  | Wbc | White blood cell count |

**Supplementary Figure 1:**


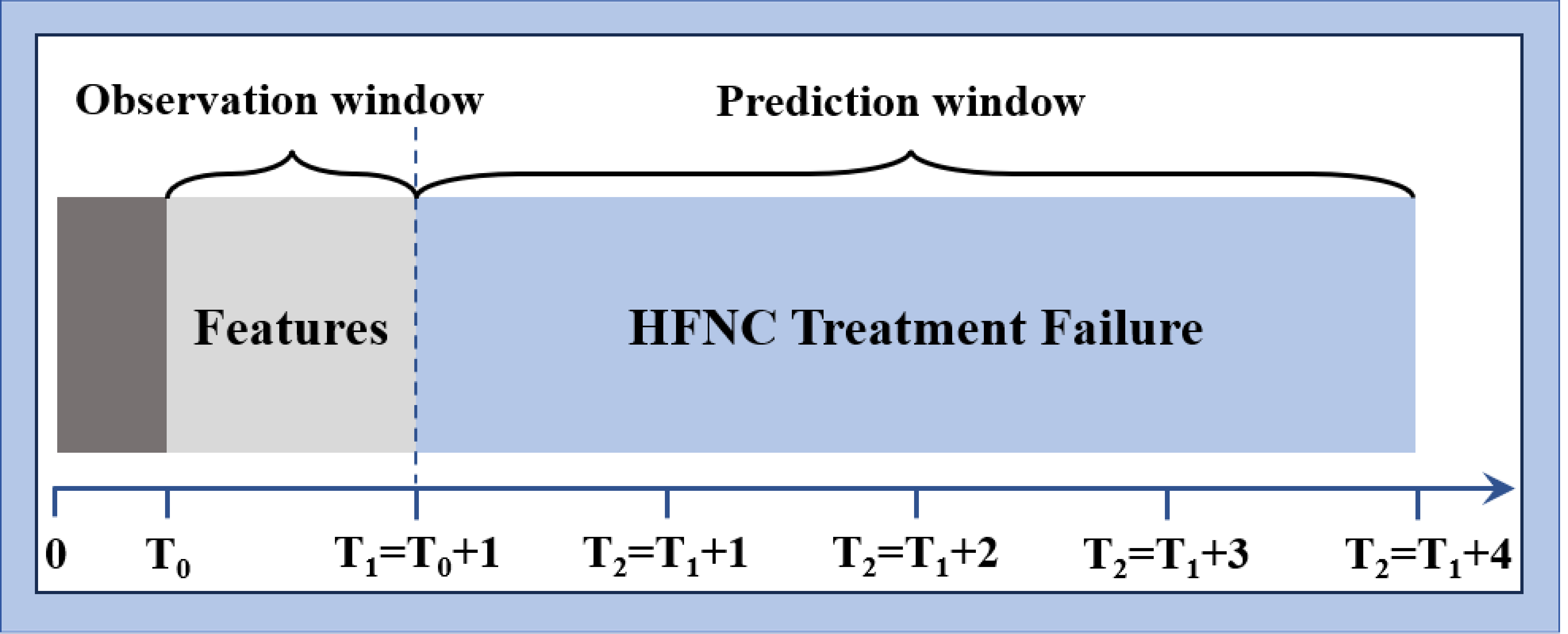


Supplementary Figure 1. Schematic timeline illustrating observation and prediction windows in HFNC failure prediction. Clinical features collected during the observation window are used to predict the risk of HFNC treatment failure in the subsequent prediction window. This diagram corresponds to the temporal design described in the main text and supports understanding of the model’s dynamic prediction mechanism.
